# Genome-mined endolysin LysMD30 in combination with colistin: synergistic antimicrobial and antibiofilm activities against *Vibrio parahaemolyticus*

**DOI:** 10.1128/aem.00221-26

**Published:** 2026-06-12

**Authors:** Tao Wang, Kaiyuan Huang, Xinxin Yang, Hao Ding, Qiong Liu, Bowen Liu, Maoda Pang, Huiling Li

**Affiliations:** 1 School of Food and Biological Engineering, Jiangsu University 506405, Zhenjiang, China; 2 Key Laboratory of Food Quality and Safety of Jiangsu Province-State Key Laboratory Breeding Base, Institute of Food Safety and Nutrition, Jiangsu Academy of Agricultural Sciences 651111 , Nanjing, China; 3 College of Light Industry and Food Science, Nanjing Forestry University 74584, Nanjing, China; 4 College of Veterinary Medicine, Yangzhou University 614704 , Yangzhou, China; 5 Hainan Hospital of the General Hospital of the People’s Liberation Army of China 623336 , Sanya, China; University of Georgia Center for Food Safety, Griffin, Georgia, USA

**Keywords:** *Vibrio*, endolysin, colistin, synergistic bactericidal effect, biofilm elimination

## Abstract

*Vibrio parahaemolyticus* is a foodborne pathogen that threatens seafood safety globally. Endolysins are promising antibacterial candidates with rapid action and low resistance induction. Through genome mining of 163 *Vibrio* phages, we identified 168 potential endolysin genes and expressed the first *Vibrio*-phage-derived L-alanyl-D-glutamate peptidase, LysMD30, in *Pichia pastoris*. Although LysMD30 exhibited peptidoglycan hydrolytic activity, it lacked an intrinsic bactericidal activity. However, it displayed broad-spectrum synergy with colistin against pathogenic *Vibrio* species (*V. parahaemolyticus*, *V. harveyi*, and *V. alginolyticus*), yielding fractional inhibitory concentration index values lower than 0.5. This synergism was marked by a fourfold reduction in the minimum inhibitory concentration of colistin, decreasing from 16–32 μg/mL to 4–8 μg/mL across all tested strains. The bactericidal kinetics indicated that the combination triggered rapid lysis within 5 min, and the majority of turbidity reduction occurred within 20 min. Moreover, the combination effectively eradicated biofilms on both polystyrene and stainless steel surfaces within 1 h, reducing the viable bacterial counts by >3  log units. In the *Galleria mellonella* infection model, the combination provided 100% protection against lethal *Vibrio* challenge while confirming its excellent *in vivo* safety. Furthermore, LysMD30 exhibited robust biochemical stability—maintaining 36.96% activity at 85°C, over 90% activity at pH 5.0–8.0, and exceeding 60% activity at 800 mM NaCl—while effectively delaying the emergence of colistin resistance in *V. parahaemolyticus* during serial passage. In summary, LysMD30 is a newly characterized and stable enzyme that synergizes powerfully with colistin and represents a promising biocontrol agent for *Vibrio* control in food and aquaculture.

**IMPORTANCE:**

*Vibrio* pathogens pose a severe threat to seafood safety and aquaculture. Conventional intervention strategies often face significant limitations: physical treatments can adversely affect the organoleptic and nutritional quality of seafood, whereas chemical agents carry the risk of toxic residues and environmental pollution. Furthermore, the escalating antimicrobial resistance of *Vibrio* and its capacity to form biofilms on food-contact surfaces exacerbate these challenges. This study identified LysMD30 as the first *Vibrio*-phage-derived L-alanyl-D-glutamate peptidase that exerts a potent synergistic bactericidal effect with colistin. This combination significantly reduced the required antibiotic dosage, rapidly eliminated both pathogenic *Vibrio* planktonic cells and biofilms, and demonstrated excellent *in vivo* safety. Our findings expand the repertoire of *Vibrio*-targeting biocontrol agents and provide an eco-friendly and efficient strategy for *Vibrio* control in food processing and aquaculture, offering robust technical support for safeguarding seafood safety and public health.

## INTRODUCTION

*Vibrio parahaemolyticus*, a typical halophilic Gram-negative pathogen, is the predominant foodborne threat associated with aquatic products (1). Currently, conventional intervention strategies encompassing physical treatments (e.g., thermal processing and freezing) and chemical disinfectants (e.g., chlorine-based agents and peracetic acid) exhibit significant constraints. Thermal methods often compromise the organoleptic properties and nutritional quality of fresh seafood, while these chemical disinfectants entail risks of toxic residues, equipment corrosion, and environmental pollution (2). Furthermore, the recalcitrance of *V. parahaemolyticus* to sanitization is exacerbated by its capacity to form robust biofilms on various food contact surfaces (3). These microbial communities, encapsulated within extracellular polymeric substances, demonstrate heightened tolerance to conventional sanitizers and facilitate persistent colonization within processing facilities, thereby increasing the risk of recurrent contamination and sanitization failure (4). Coupled with the increasing prevalence of multidrug-resistant strains driven by antibiotic misuse, the complexity of food safety management and clinical intervention has intensified significantly (5). Consequently, the development of novel, efficacious, and biosafe alternatives for controlling *V. parahaemolyticus* biofilms is of paramount importance.

Endolysins are a class of cell wall hydrolases that are encoded by double-stranded DNA phages (6). Compared to traditional antibacterial agents, endolysins provide significant advantages, including potent activity against persistent and drug-resistant bacteria, versatile host specificity, and feasibility for heterologous expression (7, 8). In addition, compared with bacteriophages, endolysins exhibit distinct advantages, including broader antibacterial spectra, more straightforward purification and standardization, and negligible risk of horizontal gene transfer (9, 10). More importantly, endolysins specifically target the highly conserved peptidoglycan scaffold in the bacterial cell wall. The structural conservation of this target greatly reduces the probability of bacteria developing resistance through mutations (11, 12), making them promising novel antibacterial agents. However, the complex outer membrane (OM) of Gram-negative bacteria acts as a natural physical barrier, hindering the contact between exogenous endolysins and internal peptidoglycans (13). This barrier greatly limits the direct bactericidal activity of endolysins against Gram-negative bacteria.

To circumvent this bottleneck, researchers have proposed synergistic strategies that combine endolysins with outer membrane permeabilizers (OMPs) (such as EDTA, cationic peptides, or specific antibiotics, etc.) (13, 14). OMPs disrupt the integrity of the outer membrane, creating channels for endolysins to enter the periplasmic space, thereby hydrolyzing peptidoglycans and inducing bacterial lysis. For example, Wang et al. (15) found that the endolysin Lysqdvp001 exhibited significant lytic activity against *V. parahaemolyticus* ATCC 17802 when supplemented with EDTA, and its combination with ε-polylysine effectively reduced the pathogen load in food (16); the combined application of Lyz_V_pgp60 and gentamicin also significantly improved the killing efficacy against *V. parahaemolyticus* (17). As a typical cationic polypeptide antibiotic, colistin destabilizes the outer membrane by competitively displacing divalent cations within lipopolysaccharides. Unlike EDTA or ε-polylysine, colistin confers a dual-functional advantage, acting as both a potent bactericidal agent and an effective OMP. This synergistic effect can markedly reduce the required colistin dosage, thereby alleviating its inherent toxicity while sustaining high efficacy against drug-resistant pathogens (18). Studies have shown that the bactericidal efficiency of the *Acinetobacter baumannii* endolysin LysAB1245, combined with colistin, is increased by 3 log units compared to that of colistin alone (18); whereas *Escherichia coli* endolysin EC340 can reduce the effective concentration of colistin to one-fourth of the original (19). These synergistic strategies not only enhance the lytic effect but also exert the additive effect of endolysins, destroying the cell wall to promote antibiotic penetration, providing a new paradigm for the mitigation of Gram-negative bacteria.

In this study, a genome mining strategy was employed to systematically identify and screen for novel *Vibrio* phage endolysins. Endolysin LysMD30 was produced using the *Pichia pastoris* heterologous expression system, and its enzymatic properties and physicochemical stability were systematically analyzed. Based on this, the synergistic antibacterial effect, bactericidal kinetics, and biofilm elimination ability of LysMD30 combined with colistin against *V. parahaemolyticus* were investigated. Finally, *in vivo* biosafety and therapeutic effectiveness were evaluated using the *Galleria mellonella* infection model. The results of this study aimed to enrich the *Vibrio* endolysin resource library and provide efficient, environmentally friendly antibacterial agents and technical support for the prevention and control of *Vibrio* infections in food processing, aquaculture, and public health.

## MATERIALS AND METHODS

### Strains and plasmids

The *P. pastoris* expression system was used in this study. *P. pastoris* GS115 served as the host strain, and pPIC9K (Invitrogen, USA) was used as the expression vector. All bacterial strains used in the experiments, including *Vibrio*, *Salmonella*, *E. coli*, and *Listeria monocytogenes,* were either purchased from standard collections or isolated and preserved in the laboratory (Table S1).

### Mining and bioinformatics analysis of endolysins

*Vibrio* phage genome sequences were retrieved from the NCBI database, and the encoded protein sequences were extracted. Following the established classification of endolysin families (N-acetyl-β-D-glucosaminidase, N-acetyl-β-D-muramidase, transglycosylase, N-acetylmuramoyl-L-alanine amidase, L-alanyl-D-glutamate endopeptidase, and interpeptide bridge endopeptidase) (20), potential endolysin candidate sequences were screened. Simultaneously, the amino acid sequences of the reported *Vibrio* endolysins were retrieved from the literature sources detailed in Table S2. Multiple sequence alignment was performed using the MUSCLE algorithm in Geneious Prime (version 2025.02) (Biomatters Ltd., New Zealand), and a phylogenetic tree was constructed using Geneious Tree Builder (with default parameters). The phylogenetic tree was visually refined using iTOL (version 7.4) (21). A heatmap illustrating the amino acid sequence similarity was generated using ClustVis (version 2.0) (22). Conserved domains of the candidate endolysins were analyzed, and functional domains were identified using the NCBI Conserved Domains database (23). Physicochemical properties, including molecular weight and isoelectric point, were predicted using ProtParam (https://web.expasy.org/protparam/). Furthermore, the tertiary structure of the protein was predicted using the Phyre2 online platform (24) in “Normal” modeling mode to evaluate its structural and functional characteristics.

### Gene synthesis, expression, and purification of endolysins

Based on the codon bias of *P. pastoris*, the gene sequences of six candidate *Vibrio* endolysins were codon-optimized, followed by *de novo* gene synthesis by the GenScript Biotech Corporation (China). The synthesized gene fragments were cloned into the expression vector pPIC9K (at EcoRI and NotI sites) via homologous recombination. The recombinant plasmids were linearized with *Sal* I and electrotransformed into *P. pastoris* GS115 cells. Transformants were screened on YNB plates, and positive clones were confirmed by PCR. Verified positive clones were inoculated into BMGY medium and cultured at 30°C and 220 rpm for 12 h. The cells were collected by centrifugation and resuspended in BMMY induction medium. Methanol was added at 1% (vol/vol) every 24 h to maintain induction for 72 h. Then, the expressed products were purified via Ni²^+^-NTA affinity chromatography, utilizing stepwise elution with a gradient of imidazole (20–500 mM). Eluted fractions were collected and analyzed by SDS-PAGE for purity, and high-purity fractions were selected for ultrafiltration concentration and subsequent experiments.

### Analysis of hydrolytic and antibacterial activities of endolysin lysMD30

First, a peptidoglycan plate containing *V. parahaemolyticus* ATCC 33847 was prepared, as described by Bai et al. (25), to detect the hydrolytic activity of LysMD30. Briefly, 10 μL of LysMD30 (128 μg/mL) was spotted onto a peptidoglycan plate, and the formation of a transparent hydrolysis zone was observed after incubation at 37°C for 12 h. Subsequently, the double-layer plate method was employed to evaluate its antibacterial activity. *V. parahaemolyticus* ATCC 33847 was cultured to the logarithmic growth phase (OD_600_ between 0.4 and 0.6). A 100 μL aliquot of the bacterial suspension was mixed with 4 mL of LB medium containing 0.6% agar and poured onto the surface of solid LB plates. After the bacterial layer solidified, 10 μL LysMD30 (128 μg/mL) was spotted, and the plate was incubated at 37°C for 12 h to observe inhibition zones. To provide a more sensitive and quantitative evaluation, strain ATCC 33847 (approximately 2 × 10^7^ CFU/mL) was incubated with LysMD30 (final concentration of 128 μg/mL) in PBS at 37°C for 1 h. After incubation, the mixtures were serially diluted and plated on LB agar to determine the number of surviving colonies.

### Synergistic lytic spectrum analysis of endolysin LysMD30 with colistin

A total of 20 bacterial strains were selected to evaluate the synergistic lytic activity of LysMD30 using colistin as an OMP. These included 12 *Vibrio* strains (six *V. parahaemolyticus*, four *V. harveyi*, and two *V. alginolyticus* strains), three *Salmonella* strains, three *E. coli* strains, and two *L. monocytogenes* strains. The test strains were cultured to the logarithmic growth phase, washed twice with PBS, and resuspended to an OD_600_ of approximately 0.6. The microplate method was used for quantification: 160 μL of bacterial suspension was added to each well, followed by 20 μL of colistin (final concentration 128 μg/mL) and 20 μL of LysMD30 (final concentration 32 μg/mL). An equal volume of PBS served as the negative control. Changes in the OD_600_ were dynamically monitored at 37°C using a microplate reader (Tecan Infinite M200 PRO, Switzerland). After incubation for 1 h, the lytic efficiency against various strains was determined by calculating the percentage reduction in blank-corrected OD_600_ values. All experiments were performed with three technical and three independent biological replicates.

The double-layer plate method was used to evaluate the synergistic antibacterial effects of LysMD30 and colistin. Logarithmic-phase *V. parahaemolyticus* ATCC 33847 bacterial solution (100 μL) was mixed with 0.6% LB agar and poured onto solid LB plates to prepare bacterial lawns. After solidification, 10 μL of colistin (4 μg/mL), LysMD30 (64 μg/mL), their mixture (LysMD30 64 μg/mL + colistin 4 μg/mL), and the PBS control were spotted. After incubation at 37°C for 12 h, the size of the inhibition zones across each treatment group was observed and compared.

### Determination of minimum inhibitory concentration and minimum bactericidal concentration

Following the method of Ding et al. (26), with slight modifications, the minimum inhibitory concentration (MIC) and minimum bactericidal concentration (MBC) of LysMD30 against *V. parahaemolyticus* ATCC 33847, *V. harveyi* 24VH06, and *V. alginolyticus* 22VA32 were determined. Each test strain was cultured to the logarithmic growth phase (OD_600_ between 0.4 and 0.6) and diluted 1:100 with fresh LB medium. Using the microbroth dilution method, 100 μL of the bacterial suspension, 50 μL of LysMD30 (0–64 μg/mL), and 50 μL of colistin (0–32 μg/mL) were sequentially added to a 96-well plate. OD_600_ was measured after incubation at 37°C for 24 h. MIC was defined as the lowest concentration that inhibited visible growth (blank-corrected OD_600_ < 0.01). The synergistic effect between LysMD30 and colistin was quantified by fractional inhibitory concentration index (FICI). The FICI was calculated according to the following formula: FICI = (MIC_A in combination_/MIC_A alone_) + (MIC_B in combination_/MIC_B alone_), where A represents LysMD30 and B represents colistin. Since LysMD30 alone exhibited no detectable MIC at concentrations up to 256 μg/mL, a value of 512 μg/mL was adopted as its MIC for FICI calculations. The interaction was interpreted as synergy (FICI **≤** 0.5), additivity (0.5 < FICI **≤** 1.0), indifference (1.0 < FICI **≤** 4.0), or antagonism (FICI > 4.0) (18). To determine the MBC in LB medium, bacterial suspensions from wells adjacent to the MIC, positive controls, and negative controls were serially diluted 10-fold with PBS and spread on LB plates for colony counting. MBC was defined as the lowest concentration that killed ≥99.9% of the initial inoculum. To determine the MBC in PBS, the checkerboard method was employed by replacing the LB medium with PBS. After incubation for 1 h, viable bacteria were quantified as described above.

### Evaluation of synergistic bactericidal kinetics of LysMD30 with colistin

Logarithmic-phase *V. parahaemolyticus* ATCC 33847, *V. harveyi* 24VH06, and *V. alginolyticus* 22VA32 were washed twice and resuspended in PBS to an OD_600_ of 0.6. The microplate method was used to monitor changes in absorbance: 160 μL of bacterial suspension was added to a 96-well plate, followed by 20 μL of LysMD30 (final concentrations: 4, 8, 16, 32, 64, and 128 μg/mL) and 20 μL of colistin (final concentration: 128 μg/mL). PBS served as the control. OD_600_ was measured dynamically every 5 min for 1 h at 37°C. The synergistic effect was further evaluated using time-kill curves; the bacterial density was adjusted to 2 × 10^7^ CFU/mL. Then, 100 μL of the bacterial solution and 100 μL of the treatment solution (colistin 4 μg/mL, LysMD30 64 μg/mL, their mixture, or PBS) were incubated at 37°C. To quench the antimicrobial activity at specific time points, samples were collected at 10 min and 1 h, followed immediately by a 10-fold serial dilution in sterile PBS. The samples were then further serially diluted and plated for viable count determination.

### Scanning electron microscopy of bacterial morphology

Logarithmic-phase *V. parahaemolyticus* ATCC 33847 was adjusted to 1 × 10^8^ CFU/mL. Colistin (2 μg/mL), LysMD30 (32 μg/mL), their mixture, and PBS control were added to the suspension. After incubation at 37°C for 1 h, bacteria were collected by centrifugation (5,000 × *g*, 5 min) and washed three times. The pellet was fixed overnight with 2.5% (vol/vol) glutaraldehyde at 4°C. After rinsing, the samples were dehydrated using a graded ethanol series (30%, 50%, 70%, 90%, and 100%). Finally, after critical point drying and gold sputtering, cell morphology was visualized using a scanning electron microscope (Zeiss, EVO-LS10, Germany).

### Evaluation of the ability of LysMD30 to eliminate *V. parahaemolyticus* biofilms

Determination of biofilm elimination by crystal violet staining: the biofilm elimination efficacy of LysMD30 and colistin against *V. parahaemolyticus* ATCC 33847 was evaluated using the crystal violet staining method, as previously described (27), on both polystyrene and stainless steel surfaces. For polystyrene surfaces, logarithmic-phase strains were inoculated into 96-well plates at a ratio of 1:100 (200 μL of LB medium per well) and statically cultured at 37°C for 24 h to construct biofilms. The culture supernatant was discarded, and the plate was gently washed three times with PBS to remove planktonic bacteria. The following treatment solutions were added: colistin (4 μg/mL), LysMD30 (64 μg/mL), their mixture (LysMD30 64 μg/mL + colistin 4 μg/mL), and PBS control, and incubated at 37°C for 1 h. After treatment, the plates were washed with PBS, fixed with methanol for 15 min, and stained with 0.1% (wt/vol) crystal violet for 10 min. After removing the excess dye, 95% ethanol was added to dissolve the bound crystal violet, and the absorbance (OD) value was measured at 595 nm using a microplate reader. The assay was conducted in parallel using 24-well plates containing sterile stainless steel coupons (1 cm × 1 cm × 0.6 mm) to evaluate surface-dependent effects. Each group had eight replicate wells, and the experiment was independently repeated three times.

Evaluation of viable bacterial counts in biofilms by the plate counting method: to evaluate the survival status of bacteria in biofilms after treatment, the plate counting method was used for detection. The treatment process for biofilms was the same as the step of determination by crystal violet staining. After incubation for 1 h, the treatment solution was removed, and the biofilm was resuspended in PBS via vigorous repeated pipetting and mechanical scraping of the well bottom. The bacterial suspension was serially diluted 10-fold and spread onto LB agar plates. After culturing at 37°C, colony counting was performed. Each group contained five parallel wells, and the experiment was independently repeated three times.

Observation of biofilm structure by two-photon laser confocal microscopy: to evaluate the effect of LysMD30 and colistin on the three-dimensional structure and cell viability of biofilms, confocal imaging technology was used. Logarithmic-phase *V. parahaemolyticus* ATCC 33847 was inoculated into 12-well plates containing cell-climbing slices at a ratio of 1:100 (1.5 mL LB medium per well) and statically cultured for 24 h to form biofilms. After washing with PBS, each group of treatment solutions was added at the concentration described in the step of determination by crystal violet staining and incubated for 1 h. Staining was performed using the Live/Dead BacLight Bacterial Viability Kit (Biotopped, China) (SYTO 9 and PI mixed in equal volume, incubated in the dark for 15 min), and excess dye was rinsed off with PBS. Finally, a two-photon laser confocal microscope (Leica STELLARIS 8 DIVE, Germany) was used for three-dimensional imaging to analyze the thickness, structural integrity, and distribution of live/dead cells of the biofilm.

### Analysis of bactericidal stability of endolysin LysMD30

To further evaluate the environmental stability of LysMD30, the effects of temperature, pH, and salt concentration on bactericidal activity were determined. For temperature stability determination, *V. parahaemolyticus* ATCC 33847 was cultured to the logarithmic phase (OD_600_ between 0.4 and 0.6). Bacteria were collected by centrifugation, washed, and resuspended in PBS, and the OD_600_ was adjusted to 0.6. LysMD30 was incubated in a water bath at 4°C–85°C for 1 h and then returned to room temperature. Then, 160 μL of bacterial suspension, 20 μL of pre-treated enzyme solution (final concentration 32 μg/mL), and 20 μL of colistin (final concentration 128 μg/mL) were sequentially added to a 96-well plate, and OD_600_ was measured after reaction at 37°C for 1 h. For determination of pH and salt concentration, *V. parahaemolyticus* ATCC 33847 was cultured to logarithmic growth phase (OD_600_ between 0.4–0.6), bacteria were collected by centrifugation and resuspended in buffers with different pH (2–13) or different NaCl concentrations (0–800 mM). The bacterial suspension (160 μL), 20 μL LysMD30 (final concentration 32 μg/mL), and 20 μL colistin (final concentration 128 μg/mL) were added to a 96-well plate, and an equal volume of PBS was used instead of the enzyme solution as a control. OD_600_ was measured after the reaction at 37°C for 1 h. All experiments were performed with three technical replicates and independently repeated three times. The relative activity of each treatment group was calculated by taking the highest activity under optimal conditions of 100%.

### Protective efficacy and safety evaluation of LysMD30 in *G. mellonella* infection model

A *G. mellonella* infection model was used to evaluate the *in vivo* antibacterial activity and safety of LysMD30. Healthy *G. mellonella* with a body length of approximately 3 cm, acquired from Payuan company in Shanghai, were randomly divided into four groups: colistin treatment group (2 μg/mL), LysMD30 treatment group (32 μg/mL), combined treatment group (LysMD30 32 μg/mL + colistin 2 μg/mL), and PBS control group. *V. parahaemolyticus* ATCC 33847 was cultured to the logarithmic growth phase, washed, and resuspended, and the bacterial solution concentration was adjusted to 1 × 10^4^ CFU/mL. Modeling was performed by injecting 10 μL of the bacterial suspension into the left hind leg coxa of *G. mellonella* using a microsyringe. One hour after the infection, 10 μL of the corresponding treatment solution was injected into the right hind leg. Meanwhile, control groups only injected with colistin, LysMD30, the combined treatment group, or PBS were used to evaluate the *in vivo* safety. *G. mellonella* in each group were incubated at 37°C in the dark, their survival status was continuously recorded every 12 h for 72 h, and survival curves were drawn.

### Assessment of LysMD30 on the evolution of colistin resistance in *V. parahaemolyticus*

An *in vitro* serial passage assay was conducted to evaluate the impact of LysMD30 on the evolution of colistin resistance in *Vibrio parahaemolyticus*. Sub-MIC of colistin, used either alone (8 μg/mL) or in combination with LysMD30 (16 μg/mL), served as the selective pressure. Briefly, the bacterial suspension was inoculated into LB broth containing the respective agents and incubated at 37°C with shaking (180 rpm) for 18–24 h. Subsequently, the cultures were transferred into fresh media containing the same drug concentrations for 30 consecutive generations, with bacterial samples collected every three generations. The MICs of colistin, both as a monotherapy and in combination with LysMD30, were determined for each generation using the broth microdilution method. In the synergistic MIC assays, the concentration of LysMD30 was 32 μg/mL, while colistin was subjected to twofold serial dilutions ranging from 4 to 128 μg/mL. The evolutionary trajectory of resistance was evaluated by analyzing the dynamic shifts in MIC values across generations to determine the inhibitory effect of LysMD30 on colistin-induced resistance evolution.

### Statistical analysis

All data were statistically analyzed using SPSS Statistics software (version 22.0), and the experimental results are expressed as “mean ± Standard Deviation.” To meet the requirements of normality and homoscedasticity, viable bacterial counts (CFU/mL) were subjected to log_10_ transformation, while MIC data were log_2_-transformed before statistical analysis. One-way analysis of variance was used to evaluate differences between groups, and Tukey’s test or Dunnett’s T3 test was used for post hoc testing. *P* < 0.05 was considered statistically significant, and *P* < 0.01 was considered extremely statistically significant. All data graphs were drawn using the GraphPad Prism software (version 10.0).

## RESULTS

### Identification and bioinformatics analysis of *Vibrio* phage-derived endolysins

**Fig 1:**
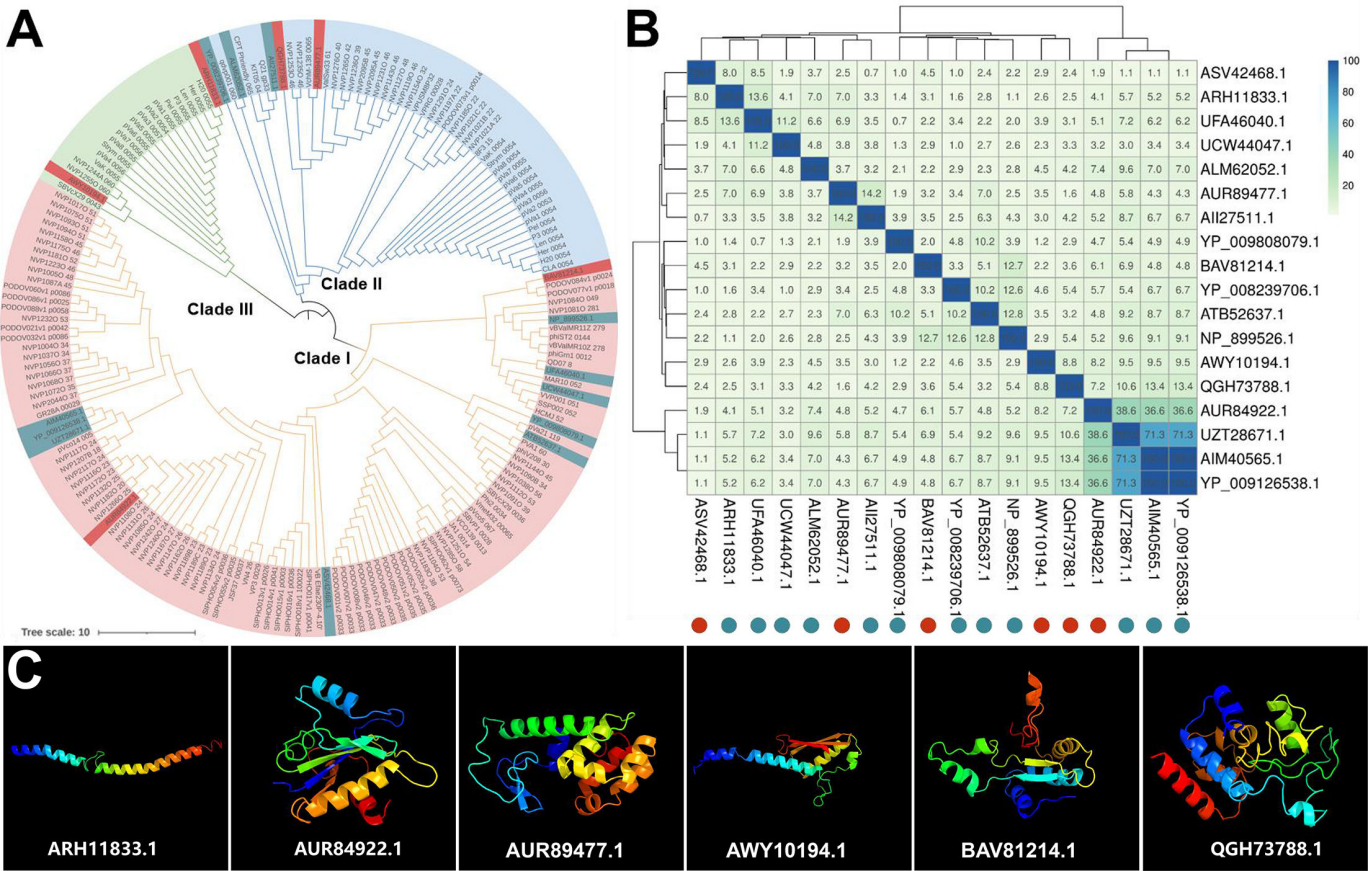
Phylogenetic analysis, sequence similarity, and tertiary structure prediction of *Vibrio* phage endolysins. (**A**) Phylogenetic tree constructed from 180 *Vibrio* endolysin sequences. Cyan clades represent previously reported endolysins, and red clades indicate candidate endolysins screened in this study. (**B**) Heatmap illustrating the amino acid sequence similarity of 18 representative endolysins, including 12 known endolysins (cyan markers) and 6 candidate endolysins (red markers). (**C**) Predicted tertiary structure models of the six candidate endolysins.

A total of 180 endolysin sequences were identified in 163 *Vibrio* phage genomes retrieved from the NCBI database. Among them, 168 were newly predicted endolysins without prior functional characterization (Table S3), whereas the remaining 12 endolysins were previously reported (Table S2). These endolysins primarily belong to families that include N-acetylmuramoyl-L-alanine amidase, L-alanyl-D-glutamate endopeptidase, and transglycosylase. Their amino acid sequence lengths ranged from 67 to 367 amino acids, with predicted molecular weights spanning 7.48 to 41.36 kDa. Phylogenetic analysis categorized these endolysins into three main evolutionary clades (I–III). The 12 reported endolysins (Table S2) were concentrated in clades I (9) and II (3), whereas no distribution was observed in clade III (Fig. 1A). Based on this phylogenetic distribution, we screened candidate endolysins that were distantly related to known sequences to identify structurally novel enzymes. By comprehensively considering phylogenetic position, functional annotation, and molecular weight, six previously uncharacterized endolysins (ARH11833.1, AUR84922.1, AWY10194.1, BAV81214.1, QGH73788.1) were selected for recombinant expression. Their amino acid lengths ranged from 126 to 189 aa, with predicted molecular weights between 14.23 and 21.91 kDa (Table S3). Sequence alignment analysis was performed to evaluate the novelty of the selected endolysins. As shown in Fig. 1B, the amino acid sequence similarity between these six candidates and all reported endolysins was low, with the highest similarity reaching only 38.6% (between AUR84922.1 and UZT28671.1). Tertiary structure prediction further indicated that all candidate endolysins existed in monomeric form, exhibiting significant structural diversity (Fig. 1C).

### Expression, purification, and activity determination of *Vibrio* phage-derived endolysins

**Fig 2:**
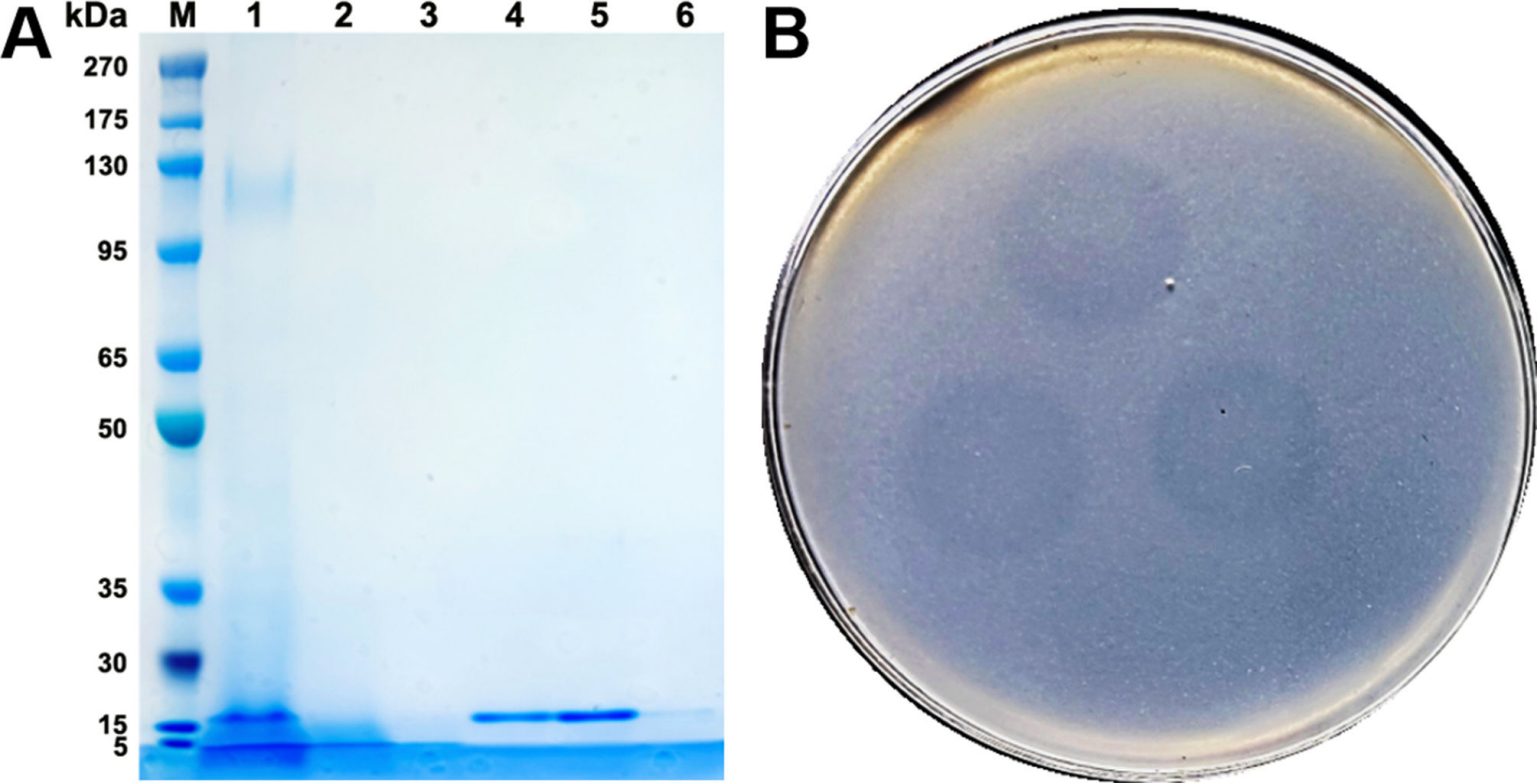
SDS-PAGE analysis (**A**) and peptidoglycan hydrolytic activity (**B**) of LysMD30. (**A**) Lane M: protein molecular weight marker; lane 1: flow-through; lanes 2–6: elution fractions with 20, 50, 100, 200, and 500 mM imidazole, respectively. (**B**) The three clear zones represent triplicate spots of LysMD30.

To evaluate the function of the candidate genes, six sequences were recombinantly expressed in the *P. pastoris* system. AUR84922.1, BAV81214.1, and QGH73788.1 were successfully expressed. However, the expression levels of AUR84922.1 and QGH73788.1 remained insufficient for further study despite optimization. In contrast, BAV81214.1 exhibited high expression levels and was subsequently named LysMD30 for in-depth characterization. LysMD30 was purified via Ni²^+^-NTA affinity chromatography and effectively eluted at imidazole concentrations of 100 and 200 mM. The purification process yielded a protein concentration of 526 μg/mL (Fig. 2A). Sequence analysis revealed that LysMD30 is encoded by 128 amino acids, with a predicted molecular weight of 15.03 kDa and an isoelectric point (pI) of 6.03 (Table S3). This protein belongs to the L-alanyl-D-glutamate peptidase family, which contains a peptidase_M15-like domain and three key catalytic residues (R39, H62, and D71). Tertiary structure prediction revealed that LysMD30 is a monomeric protein composed of five α-helices and three β-sheets (Fig. 1C).

To verify its enzymatic function, peptidoglycan plates were prepared using thermally inactivated *V. parahaemolyticus*. The results showed that LysMD30 hydrolyzed peptidoglycan to form a clear transparent zone (Fig. 2B), confirming its peptidoglycan hydrolytic activity. However, no inhibition zone was observed when LysMD30 was directly spotted onto *V. parahaemolyticus* lawns, and no significant bactericidal activity was detected in the quantitative liquid-phase assay, indicating that this endolysin lacked the ability to directly kill intact live bacteria.

### Synergistic lytic spectrum of endolysin LysMD30 with colistin

**Fig 3:**
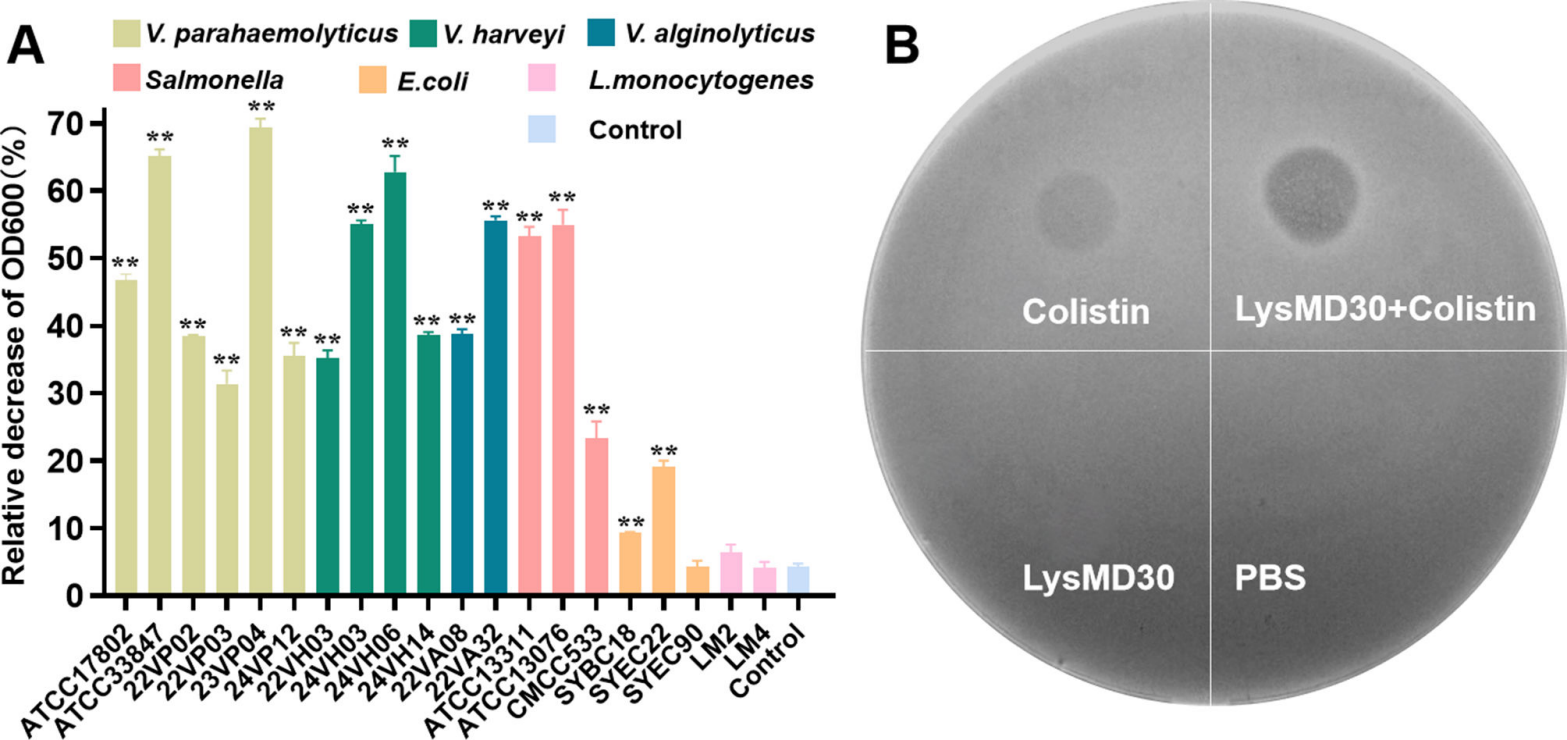
Synergistic lytic activity of LysMD30 combined with colistin. (**A**) Lytic efficiency of the LysMD30-colistin combination against 20 different strains after 60 min of treatment. (**B**) Inhibition zones formed by different treatment groups on *V. parahaemolyticus* double-layer agar plates. Due to the consistent performance of control groups across strains, *V. parahaemolyticus* ATCC 33847 was used as the representative model for statistical analysis. ** indicates a significant difference (*P* < 0.01) between the combination treatment and the PBS control group.

To evaluate the bactericidal activity of LysMD30 after bypassing the outer membrane, colistin was used as an outer membrane permeabilizer. The lytic effect of the combination was tested against 20 strains across four different genera (Fig. 3A). To clearly present the lytic efficiency across multiple strains, the reduction in OD_600_ after 60 min of treatment is shown in Fig. 3A, while the complete growth curves are provided in Fig. S1. The results showed that the combination of LysMD30 and colistin significantly reduced the OD_600_ values of all tested strains, with reductions ranging from 4.10% to 69.37% (Fig. 3A). The degradation effect on *V. parahaemolyticus* ATCC 33847, 23VP04, and *V. harveyi* 24VH06 was particularly pronounced, with an OD_600_ decrease of more than 60% (*P* < 0.01). Notably, reductions in *Salmonella* strains ranged from 23.33% to 55.01% (*P* < 0.01). In contrast, the antibacterial effect against *E. coli* strains was relatively weaker, although significant decreases were still observed in two of the three tested *E. coli* strains, namely SYBC18 and SYEC22 (*P* < 0.01). No significant antibacterial activity against the Gram-positive *L. monocytogenes* was observed. Plate antibacterial assays using *V. parahaemolyticus* ATCC 33847 as a representative model showed that compared to colistin alone, the addition of LysMD30 produced a larger and more transparent inhibition zone, indicating a significant synergistic antibacterial effect (Fig. 3B). These results demonstrated that LysMD30, when combined with colistin, can effectively lyse various pathogens beyond the *Vibrio* genus, exhibiting broad-spectrum synergistic potential.

### MIC and MBC of the synergistic effect of LysMD30 with colistin

**Fig 4:**
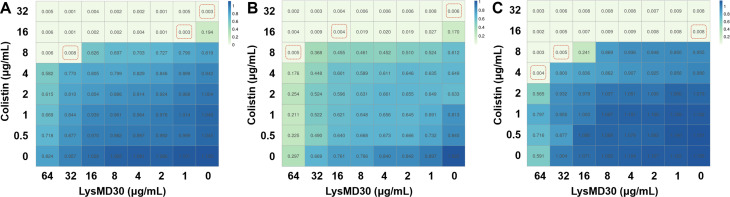
Evaluation of the synergistic antibacterial effects of LysMD30 and colistin using the checkerboard microdilution assay. Panels **A**, **B,** and **C** represent strain ATCC 33847, 24VH06, and 22VA32, respectively.

To quantitatively evaluate the synergistic antibacterial effects of LysMD30 and colistin, *V. parahaemolyticus* ATCC 33847, *V. harveyi* 24VH06, and *V. alginolyticus* 22VA32 were selected as representative strains. The MIC and MBC of the combinations were determined using the checkerboard microdilution method and further evaluated by calculating the FICI. As shown in Fig. 4, the MICs of colistin alone against the three strains were 32 μg/mL (ATCC 33847), 32 μg/mL (24VH06), and 16 μg/mL (22VA32). When combined with LysMD30, a significant synergistic effect was observed (FICI < 0.5), resulting in various effective concentration combinations. For ATCC 33847, the synergistic MIC combinations included 32 μg/mL LysMD30 + 8 µg/mL colistin (FICI = 0.313), and 1 μg/mL LysMD30 + 16 µg/mL colistin. For 24VH06, the combinations used were 64 μg/mL LysMD30 + 8 µg/mL colistin (FICI = 0.375) or 16 μg/mL LysMD30 + 16 µg/mL colistin. For 22VA32, the optimal synergistic concentrations were 64 μg/mL LysMD30 + 4 µg/mL colistin (FICI = 0.375), and 32 μg/mL LysMD30 + 8 µg/mL colistin. Notably, while maintaining the same antibacterial or bactericidal efficacy, the combination significantly reduced the effective working concentration of colistin to one-fourth of that when used alone (*P* < 0.01). Specifically, the required colistin concentration decreased from 32 to 8 μg/mL for ATCC 33847 and 24VH06, and from 16 to 4 μg/mL for 22VA32.

MBC assays in LB medium showed that the MBC values for each synergistic combination were consistent with their corresponding MIC values. Furthermore, in the non-nutritive PBS environment, the MBC values of colistin against ATCC 33847, 24VH06, and 22VA32 were 16, 16, and 8 μg/mL, respectively. When combined with 32 μg/mL LysMD30, the MBC of colistin was further reduced to 2 μg/mL. In conclusion, the combination of LysMD30 and colistin showed significant synergistic antibacterial activity against the above *Vibrio* species, which could greatly improve the sensitivity of bacteria to colistin. Therefore, 32 μg/mL LysMD30 + 2 µg/mL colistin was selected as the main synergistic concentration for subsequent experiments.

### Bactericidal kinetics and scanning electron microscopy observation

**Fig 5:**
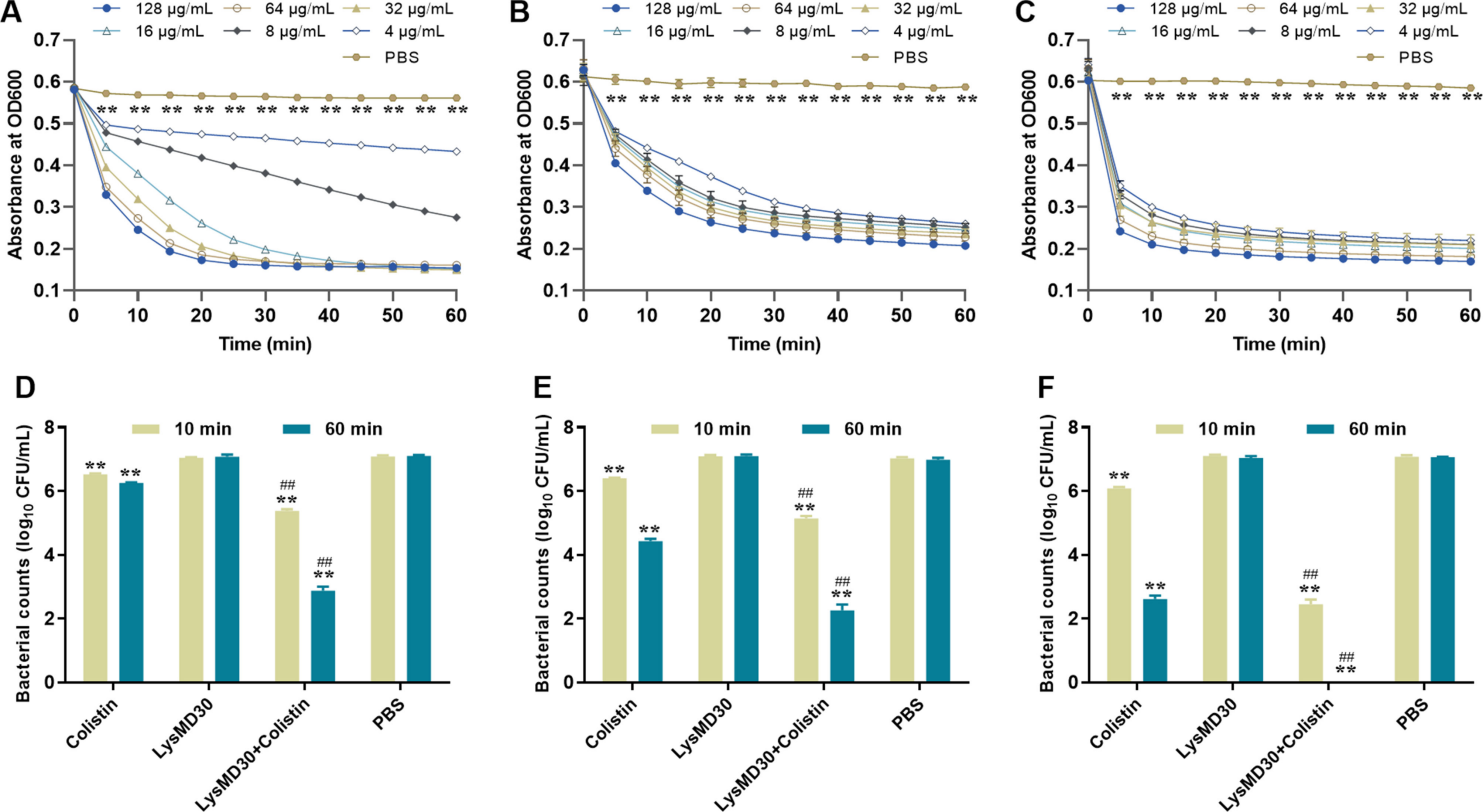
Synergistic bactericidal kinetics of LysMD30 and colistin. (**A–C**) Lytic kinetics of strains ATCC 33847 (**A**), 24VH06 (**B**), and 22VA32 (**C**); (**D–F**) viable bacterial counts of strains ATCC 33847 (**D**), 24VH06 (**E**), and 22VA32 (**F**). In panels **A–C**, ** indicates that all LysMD30 (4–128 μg/mL) colistin combination groups showed a significant difference (*P* < 0.01) compared to the PBS group at each time point; in panels **D–F**, ** indicates *P* < 0.01 when comparing treatment groups to the PBS group, while ^##^ indicates *P* < 0.01 when comparing the LysMD30-colistin combination group to the colistin group.

In the presence of colistin, the effects of different concentrations of LysMD30 on the OD_600_ of bacterial suspensions were determined within 60 min. As shown in Fig. 5A through C, the combination of LysMD30 and colistin exhibited a concentration- and time-dependent lytic effect on strains ATCC 33847, 24VH06, and 22VA32. Even at the lowest tested concentration (4 μg/mL LysMD30), the turbidity of the bacterial suspension showed a significant reduction compared to the control group (*P* < 0.01). At the highest concentration (128 μg/mL), the OD_600_ of the three strains significantly decreased to 0.15, 0.21, and 0.17, respectively, by the end of the 60-min treatment. For the time-dependent kinetics, the OD_600_ of all strains showed a statistically significant reduction within the first 5 min of treatment (*P* < 0.01), with strain 22VA32 exhibiting the most pronounced decline (OD_600_ decreased to 0.24 with a lytic rate of 0.076 units/min); bacteria continued to be lysed within 5–20 min, and the decrease in turbidity tended to be flat after 20 min. Collectively, the synergistic bactericidal effect of LysMD30 combined with colistin was statistically evident at 5 min, and most turbidity reduction was completed within 20 min.

The survival of bacteria after 10 and 60 min of combined treatment was further evaluated using the plate counting method (Fig. 5D through F). At 10 min of treatment, the viable counts in the LysMD30-colistin combination group showed significant declines, reaching 5.38, 5.15, and 2.47 log_10_ CFU/mL for ATCC 33847, 24VH06, and 22VA32, respectively (*P* < 0.01). After 60 min of treatment, the viable bacterial counts of ATCC 33847 and 24VH06 further decreased to 2.89 and 2.29 log_10_ CFU/mL, respectively, whereas no viable colonies were detected for strain 22VA32. At both time points, the bactericidal effect of the combination group was significantly better than that of the colistin alone treatment group (*P* < 0.01); no obvious bactericidal effect was observed in the LysMD30 alone and PBS control group.

**Fig 6:**
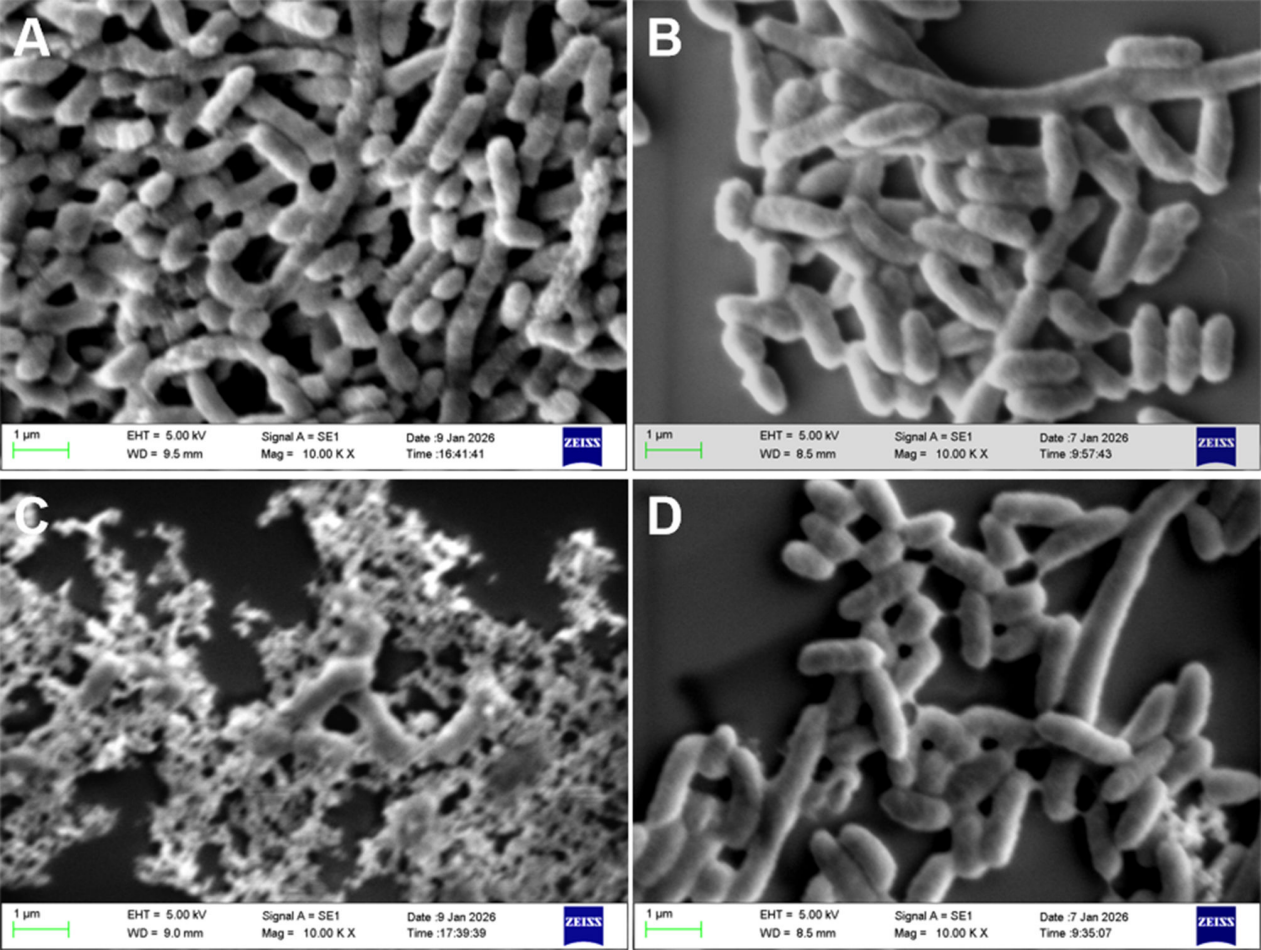
Scanning electron microscopy of *V. parahaemolyticus* under different treatments. (**A**) Colistin treatment; (**B**) LysMD30 treatment; (**C**) LysMD30 combined with colistin treatment; (**D**) PBS control.

To intuitively observe the changes in bacterial morphology, scanning electron microscopy analysis was performed using *V. parahaemolyticus* ATCC 33847 (Fig. 6). Bacteria in the PBS control group were typical intact rods that were fully shaped and closely arranged (Fig. 6D). Bacteria in the colistin-alone treatment group largely maintained a rod shape; however, scattered cells exhibited surface roughening with occasional slight shrinkage (Fig. 6A). Bacteria in the endolysin treatment group had intact morphology, similar to that in the PBS control group (Fig. 6B). In the LysMD30-colistin combined treatment group, the bacterial structure was severely damaged, most bacteria disintegrated into fragments, and only irregular cell debris remained, indicating that the two had a significant synergistic destructive effect on the bacterial cell wall (Fig. 6C).

### Biofilm elimination effect and laser confocal microscopy observation of LysMD30 with colistin

**Fig 7:**
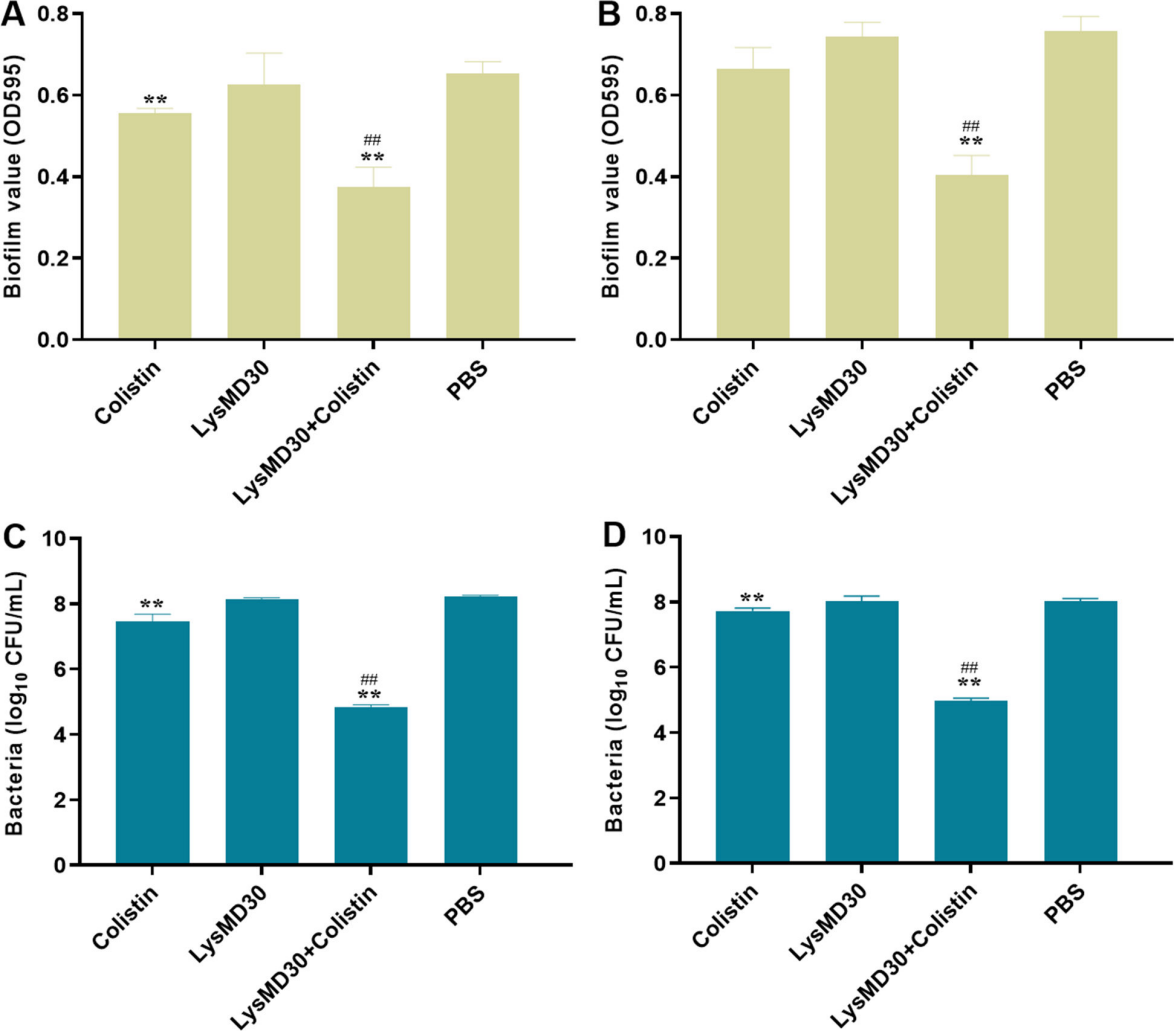
Synergistic eradication of biofilms by LysMD30 and colistin. Panels **A** and **B** show total biofilm biomass on polystyrene and stainless steel surfaces, respectively, as quantified by crystal violet staining; panels **C** and **D** show viable bacterial counts within biofilms on polystyrene and stainless steel surfaces, respectively, as determined by plate counting. ** indicates *P* < 0.01 when comparing treatment groups to the PBS group, while ^##^ indicates *P* < 0.01 when comparing the LysMD30-colistin combination group to the colistin group.

Crystal violet staining showed that on polystyrene surfaces, LysMD30 alone caused no significant biomass reduction compared to the PBS control, and colistin alone only slightly reduced biomass (*P* < 0.01). In contrast, the LysMD30-colistin combination resulted in the most pronounced decrease (from 0.65 to 0.37; *P* < 0.01; Fig. 7A). On stainless steel surfaces, the biofilm biomass in the combination group (0.40) was also significantly lower than that in all other groups (*P* < 0.01; Fig. 7B).

Viable cell counts further confirmed the synergistic effect. On polystyrene surfaces, the LysMD30-colistin combination reduced viable counts to 4.84 log_10_ CFU/mL, whereas the colistin-alone, LysMD30-alone, and PBS control groups yielded 7.51, 8.14, and 8.22 log_10_ CFU/mL, respectively (*P* < 0.01; Fig. 7C). Similarly, on stainless-steel surfaces, viable counts in the combination group (4.79 log_10_ CFU/mL) were markedly lower than those in the colistin-alone (7.73 log_10_ CFU/mL), LysMD30-alone (8.05 log_10_ CFU/mL), and PBS control (8.03 log_10_ CFU/mL) groups (*P* < 0.01; Fig. 7D). Compared to the PBS control, the combination reduced biofilm viability by more than 3 log units on both surfaces, demonstrating efficacy that was significantly superior to that of either agent alone.

**Fig 8:**
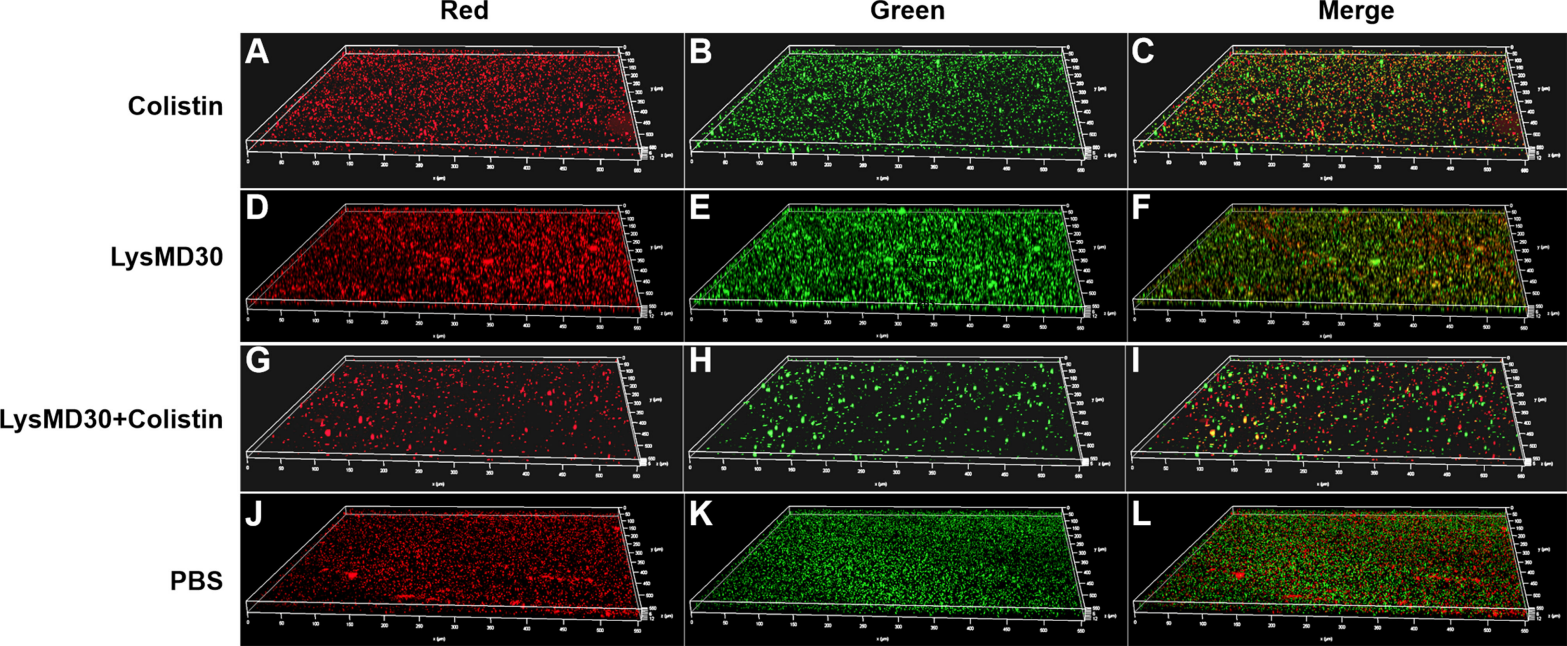
Confocal laser scanning microscopy of biofilm eradication by LysMD30 and colistin. (**A, D, G, J**) Dead bacteria were stained with propidium iodide PI (red); (**B, E, H, K**) total bacteria (live and dead) were stained with Syto 9 (green); (**C, F, I, L**) merged images of the two channels.

Confocal laser scanning microscopy intuitively illustrated the structural changes in the biofilm (Fig. 8). Biofilms in the PBS control (Fig. 8J through L) and LysMD30-only groups (Fig. 8D through F) exhibited dense and intact architectures. While the colistin-treated biofilm (Fig. 8A through C) appeared slightly loose, the architecture of the LysMD30-colistin group (Fig. 8G through I) was profoundly altered and appeared significantly sparse. The biofilm thickness decreased from approximately 12 to 5 μm, accompanied by a marked reduction in viable cell density. The above results indicate that the use of colistin or LysMD30 alone had a limited effect on the elimination of biofilms, but their combination could significantly destroy the biofilm structure, effectively kill the bacteria in the biofilm, and show an efficient synergistic elimination effect.

### Enzymatic stability analysis of endolysin LysMD30

**Fig 9:**
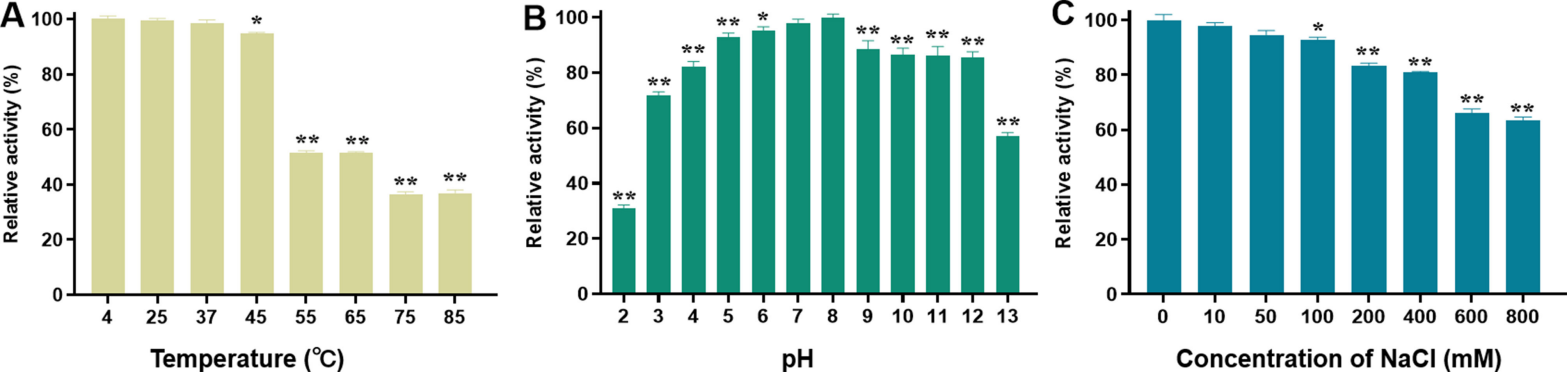
Effects of temperature (**A**), pH (**B**), and NaCl concentration (**C**) on the activity of LysMD30. * and ** indicate statistically significant differences (*P* < 0.05 and *P* < 0.01, respectively) of each group versus the group with the maximum activity.

Environmental stability of LysMD30 was assessed at various temperatures, pH values, and salt concentrations. Regarding thermal stability, the bactericidal activity remained stable after 1 h of incubation at 4°C–45°C (Fig. 9A). At 45°C, the activity decreased slightly to 94.75% of the optimal level. Although the activity declined at higher temperatures, the enzyme retained 36.96% of its activity even after treatment at 85°C, indicating robustness across a wide temperature range.

In terms of pH stability, LysMD30 exhibited maximal activity at pH 8.0 (Fig. 9B). It maintained over 90% activity between pH 5.0 and 8.0, and over 70% activity at pH 3.0 and 12.0. Even under extreme conditions (pH 2.0 and 13.0), the enzyme exhibited residual activity, demonstrating a high tolerance to acidic and alkaline environments.

Salt tolerance assays showed that LysMD30 activity remained stable between 0 and 50 mM NaCl (Fig. 9C). Activity was maintained above 80% at 100–400 mM NaCl, and even at 800 mM NaCl, the enzyme retained over 60% of its optimal activity. These findings suggest that LysMD30 possesses excellent catalytic stability in high-ionic-strength environments, providing a basis for its application in complex industrial or aquaculture settings.

### I*n vivo* protective efficacy and safety of LysMD30 in *G. mellonella* infection model

**Fig 10:**
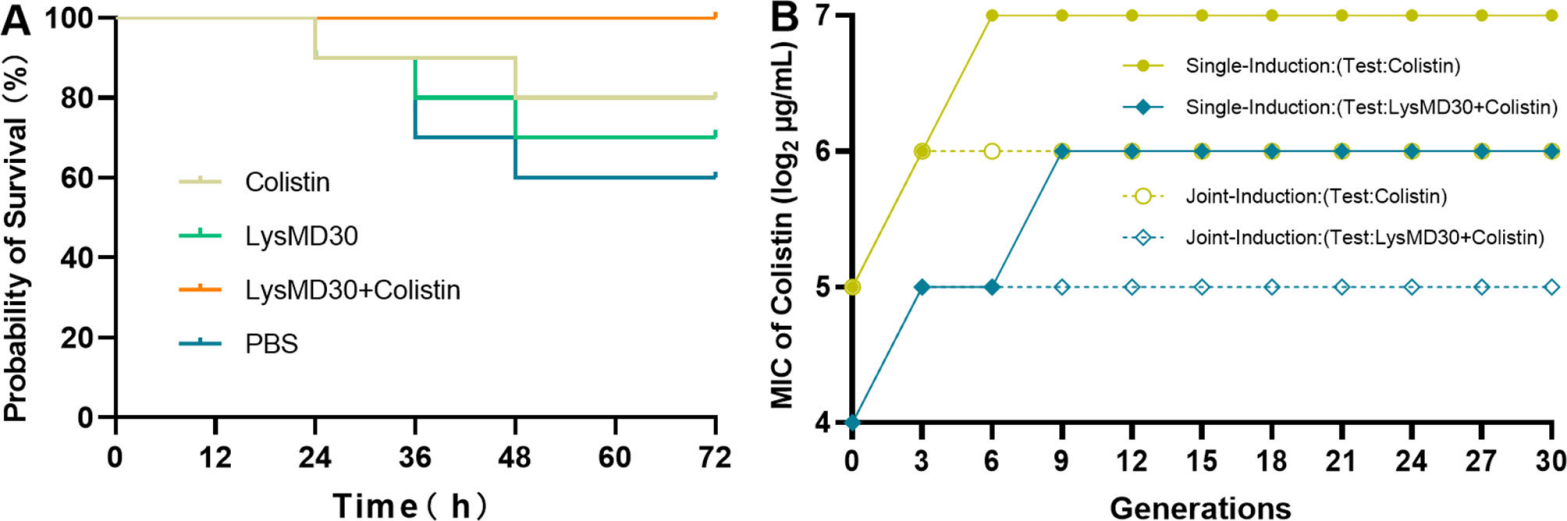
Survival curves of *G. mellonella* (**A**) and progression of colistin resistance in different treatment groups (**B**).

To verify the therapeutic potential of LysMD30 *in vivo*, we established a *G. mellonella* model infected with *V. parahaemolyticus* ATCC 33847. In the infection assays (Fig. 10A), larvae in the PBS-treated infected group began to die within 24 h, with a cumulative survival rate of 60% at 72 h. In contrast, the LysMD30 and colistin monotherapy groups showed moderate protection, with 72-h survival rates increased to 70% and 80%, respectively. Notably, the LysMD30-colistin combination showed excellent synergistic therapeutic efficacy, maintaining a 100% survival rate throughout the experiment and effectively preventing pathogen-induced lethality. Safety evaluations confirmed that non-infected larvae injected with LysMD30 alone or LysMD30-colistin combination maintained 100% survival for over 72 h, with no signs of melanization or toxicity, indicating that LysMD30 possesses excellent *in vivo* biosafety.

### Impact of LysMD30 on *in vitro* induction of colistin resistance in *V. parahaemolyticus*

To evaluate the development of resistance, *V. parahaemolyticus* ATCC 33847 was subjected to 30 continuous passages under selective pressure from colistin alone (single-induction) or the LysMD30-colistin combination (joint-induction). As shown in Fig. 10B, the parental strain (G0) exhibited an initial colistin MIC of 32 μg/mL. In the single-induction group, the colistin MIC doubled to 64 μg/mL at G3 and further peaking at 128 μg/mL from G6 to G30. When this group was tested in the presence of LysMD30 (16 μg/mL), the MIC also showed an upward trend, eventually stabilizing at 64 μg/mL from G9 onward. In contrast, the joint-induction group showed markedly delayed resistance development. When tested against colistin alone, the MIC increased to 64 μg/mL at G3, but remained completely stable through G30. When tested with the LysMD30 combination, the MIC of the joint-induction group was maintained at a low level of 32 μg/mL from G3 to G30. These findings suggest that LysMD30 not only enhances the initial susceptibility of *V. parahaemolyticus* to colistin but also effectively retards the evolution of resistance and lowers the final resistance threshold.

## DISCUSSION

*V. parahaemolyticus*, a globally significant foodborne pathogen, poses a severe threat to public health and inflicts substantial economic losses in the aquaculture industry due to its widespread transmission (1, 2, 5). Phage-derived endolysins have emerged as promising candidates for the development of novel antimicrobials because of their high efficiency, specificity, and low propensity to induce bacterial resistance (9, 27). Currently, significant progress has been achieved in researching endolysins targeting *V. parahaemolyticus*, with reported enzymes, including KVP40-LT (28), LysF23s1 (29), Lysqdvp001 (16), LysVPB (30), LysVpKK5 (31), LysVPMS1 (32), LysVPp1 (33), Lyz_V_pgp60 (17), Lyz_V_pgrp (17), Lyz_V_zlis (17), vB_VpaP_KF2_Lys (34), and MZ127813 (35). These endolysins are mainly classified into functional types, such as muramidases (e.g., LysVPp1), N-acetylmuramoyl-L-alanine amidases (e.g., LysVpKK5), and transglycosylases (e.g., LysF23s1). However, given the large *Vibrio* population under evolutionary pressure, mining for novel endolysins with unique biochemical properties and enhanced environmental adaptability remains of great biological significance.

In this study, a genome mining strategy was employed to identify 168 functionally uncharacterized endolysins from 163 *Vibrio* phage genomes, thereby significantly expanding the gene resource library for *Vibrio* endolysins. By screening candidate endolysins from phylogenetically distant branches, this strategy effectively avoids redundant research on homologs of known endolysins and increases the probability of discovering enzymes with novel characteristics. Notably, sequence alignment revealed that the amino acid sequences of LysVpKK5 (31) and Lyz_V_pgrp (17) were identical. We speculate that this duplicate naming may have resulted from literature omissions during sequence submission, highlighting the necessity of establishing standardized identification and classification procedures for endolysin development.

Regarding the selection of heterologous expression platforms, this study adopted the *P. pastoris* system instead of the traditional *E. coli* prokaryotic system. The *P. pastoris* system capitalizes on the fact that the eukaryotic cell wall lacks the endolysin substrate (peptidoglycan), thereby effectively circumventing toxicity to the host (36). Additionally, the *P. pastoris* system possesses efficient secretion capacity, enabling direct excretion of the target protein into the fermentation broth to meet the requirements of large-scale production (37). In this study, AUR84922_1, BAV81214.1 (named LysMD30), and QGH73788.1 were successfully expressed. While the expression levels of AUR84922_1 and QGH73788.1 remained low even after optimization, the yield of LysMD30 in the fermentation supernatant reached 136 μg/mL, confirming that *P. pastoris* is an ideal expression system for *Vibrio* phage endolysins.

LysMD30 was the first reported *V. parahaemolyticus* endolysin belonging to the L-alanyl-D-glutamate peptidase family, featuring a typical Peptidase_M15-like domain. Structural analysis indicated that it contains key catalytic residues (R39, H62, and D71) that cleave the peptide bond between L-Ala and D-Glu in peptidoglycan. This catalytic mechanism differs from that of the common glycoside hydrolase-type endolysins. Although LysMD30 can efficiently hydrolyze isolated peptidoglycan, similar to other *Vibrio* endolysins, such as KVP40-LT (28), Lyz_V_zlis (17), and LysVpKK5 (31), it cannot directly lyse intact viable bacteria. This is primarily attributed to the physical barrier effect of the Gram-negative outer membrane, which hinders contact between the enzyme molecules and the periplasmic peptidoglycan layer.

Colistin-mediated permeabilization effectively expanded the lytic spectrum of LysMD30 to encompass diverse Gram-negative pathogens beyond the *Vibrio* genus, such as *Salmonella* and *E. coli*. Notably, the combination was ineffective against *L. monocytogenes* because colistin specifically targets lipopolysaccharides, which are absent in Gram-positive bacteria, thereby failing to facilitate endolysin entry. Regarding *Vibrio* strains, the combination exhibited significant synergism with an FICI lower than 0.5. Colistin binds to OM lipopolysaccharides and displaces divalent cations, leading to local disruption and increased permeability, thereby creating channels through which LysMD30 can enter the periplasmic space. LysMD30 then hydrolyzes peptidoglycan, destroying the integrity of the cell wall, and ultimately inducing bacterial lysis. Bactericidal kinetic studies confirmed the high efficiency of this synergy; the treatment took effect within 5 min and reached maximal efficacy within 20 min, a reaction time significantly shorter than that of traditional antibiotics. Scanning electron microscopy intuitively revealed the endolysin-mediated cell wall disintegration process: after the peptidoglycan scaffold was cleaved by LysMD30, the imbalance of internal bacterial pressure resulted in explosive cell lysis. Similar synergistic enhancement effects have been confirmed in *Acinetobacter baumannii* endolysins, such as LysAB1245 (18) and LysMK34 (38). Notably, the efficacy of the LysMD30-colistin combination varied among different *Vibrio* strains, suggesting that the synergism between endolysins and antibiotics may exhibit strain-dependent differences.

Biofilms are dense structures that protect bacteria from antibacterial agents and are a major impediment to controlling *Vibrio* contamination during food processing (39). Traditional antibiotics and cleaning products have limited efficacy in killing biofilm-encapsulating bacteria (40). To control biofilms, chemicals such as sodium hydroxide and sodium hypochlorite are commonly used in the food industry. However, their high corrosiveness can damage equipment and materials, potentially causing environmental concerns (3). In this study, colistin alone was ineffective at eliminating biofilms. However, the combination of LysMD30 and colistin reduced the viable bacterial counts by more than 3 log units on both polystyrene and stainless steel surfaces within 1 h. These results suggest that the LysMD30–colistin combination could serve as a promising alternative antibiofilm strategy against *V. parahaemolyticus* biofilms. Furthermore, LysMD30 maintained high activity across a broad temperature (4°C–45°C), pH (3.0–12.0), and salinity (0–800 mM NaCl) ranges, indicating excellent environmental adaptability. *In vivo* experiments using the *G. mellonella* model further confirmed that LysMD30 possessed excellent safety and therapeutic potential, providing strong support for subsequent animal studies and practical applications. Moreover, the combined use of LysMD30 and colistin can reduce and delay the development of resistance in *V. parahaemolyticus* during serial passage.

Although this study confirmed that LysMD30 combined with colistin exhibits good antibacterial activity, it has several limitations: First, most experiments were performed under controlled *in vitro* conditions, which may not fully replicate the complexity of real food systems, industrial settings, and *in vivo* environments. Second, to minimize the selective pressure associated with colistin resistance, this combination should be prioritized for targeted, short-term control or specific therapeutic applications rather than routine, long-term sanitation. Third, given the broad-spectrum nature of both endolysin and colistin, their potential impact on commensal microbiota and microbial ecology remains to be elucidated. Addressing these gaps in future research will be essential to facilitate the clinical translation and practical application of LysMD30 in food safety and infection control.

In conclusion, this study began with the systematic identification of 168 endolysins from *Vibrio* phage genomes, which led to the expression and characterization of LysMD30, a first *Vibrio*-phage-derived L-alanyl-D-glutamate peptidase. The combination of LysMD30 and colistin exhibited potent synergy, reducing the colistin MIC against pathogenic *Vibrio* species to one-fourth of its original value. This synergy enabled rapid killing (≤5 min) of *V. parahaemolyticus* planktonic cells and effectively eradicated biofilms within 1 h, achieving ≥3 log reductions in viable counts. Given its broad-spectrum activity, high stability under diverse physicochemical conditions, and demonstrated *in vivo* safety, LysMD30 represents a promising biosafe candidate for controlling pathogenic *Vibrio* in food safety and public health.

## Data Availability

The amino acid sequences of the 12 previously reported and 168 candidate *Vibrio* endolysins have been deposited in the National Center for Biotechnology Information (NCBI) Protein database. The corresponding accession numbers are listed in Tables S2 and S3, respectively. All sequences can be accessed directly through the NCBI Protein database using the provided accession numbers.

## SUPPLEMENTAL MATERIAL

Supplemental material aem.00221-26-s0001.docx Tables S1 to S3; Fig. S1.

ASM does not own the copyrights to Supplemental Material that may be linked to, or accessed through, an article. The authors have granted ASM a non-exclusive, world-wide license to publish the Supplemental Material files. Please contact the corresponding author directly for reuse.
