# Supplementary material for: Genome-mined endolysin LysMD30 in combination with colistin: synergistic antimicrobial and antibiofilm activities against Vibrio parahaemolyticus
Source: Appl Environ Microbiol. 2026 Jun 12;92(7):e00221-26. doi: 10.1128/aem.00221-26 (PMC13390457; doi:10.1128/aem.00221-26)
Supplement: Supplemental material — Tables S1 to S3; Fig. S1. [file aem.00221-26-s0001.docx]

**Table S1 Strains of bacterial pathogens used in this study**

| **Number** | **Species** | **Strain name** | **GenBank**  **accession number** |
| --- | --- | --- | --- |
| 1 | *Vibrio parahaemolyticus* | ATCC17802 | / |
| 2 | *Vibrio parahaemolyticus* | ATCC33847 | / |
| 3 | *Vibrio parahaemolyticus* | 22VP02 | PZ189210 |
| 4 | *Vibrio parahaemolyticus* | 22VP03 | PZ189211 |
| 5 | *Vibrio parahaemolyticus* | 23VP04 | PZ189212 |
| 6 | *Vibrio parahaemolyticus* | 24VP12 | PZ189213 |
| 7 | *Vibrio harveyi* | 22VH03 | PZ189214 |
| 8 | *Vibrio harveyi* | 24VH03 | PZ189215 |
| 9 | *Vibrio harveyi* | 24VH06 | PZ189216 |
| 10 | *Vibrio harveyi* | 24VH14 | PZ189217 |
| 11 | *Vibrio alginolyticus* | 22VA08 | PZ189218 |
| 12 | *Vibrio alginolyticus* | 22VA32 | PZ189219 |
| 13 | *Salmonella* Typhimurium | ATCC13311 | CP009102.1 |
| 14 | *Salmonella* Enteritidis | ATCC13076 | / |
| 15 | *Salmonella* Pullorum | CMCC533 | / |
| 16 | *Escherichia coli* | SYBC18 | PV276766 |
| 17 | *Escherichia coli* | SYEC22 | PV290967 |
| 18 | *Escherichia coli* | SYEC90 | PV290968 |
| 19 | *Listeria monocytogenes* | LM2 | PV270059 |
| 20 | *Listeria monocytogenes* | LM4 | PV270060 |

**Table S2 Information of 12 publicly reported *Vibrio parahaemolyticus* endolysins**

| **Serial number** | **Name** | **NCBI Protein**  **Accession number** | **Molecular weight (kDa)** | **Sequence length (aa)** | **% Polar uncharged amino acids** | **Isoelectric point** | **Reference** |
| --- | --- | --- | --- | --- | --- | --- | --- |
| 1 | KVP40-LT | NP_899526.1 | 24.29 | 208 | 30.77% | 10.05 | [1] |
| 2 | LysF23s1 | UCW44047.1 | 22.85 | 196 | 33.16% | 9.27 | [2] |
| 3 | Lysqdvp001 | ALM62052.1 | 25.90 | 236 | 26.69% | 5.92 | [3] |
| 4 | LysVPB | UZT28671.1 | 18.14 | 163 | 28.83% | 7.12 | [4] |
| 5 | LysVpKK5 | AIM40565.1 | 18.21 | 163 | 23.93% | 6.34 | [5] |
| 6 | LysVPMS1 | YP_008239706.1 | 21.34 | 187 | 34.76% | 4.79 | [6] |
| 7 | LysVPp1 | AII27511.1 | 28.23 | 254 | 24.80% | 4.26 | [7] |
| 8 | Lyz_V_pgp60 | ASV42468.1 | 7.48 | 67 | 22.39% | 4.56 | [8] |
| 9 | Lyz_V_pgrp | YP_009126538.1 | 18.21 | 163 | 23.93% | 6.34 | [8] |
| 10 | Lyz_V_zlis | ATB52637.1 | 21.60 | 191 | 24.61% | 6.52 | [8] |
| 11 | vB_VpaP_KF2_Lys | YP_009808079.1 | 41.36 | 367 | 23.43% | 4.13 | [9] |
| 12 | MZ127813 | UFA46040.1 | 14.80 | 129 | 34.11% | 6.34 | [10] |

**Table S3 Information of 168 candidate *Vibrio* endolysins**

| **Serial number** | **NCBI Protein**  **Accession**  **number** | **Molecular weight (kDa)** | **Sequence length (aa)** | **% Acidic amino acids** | **% Basic amino acids** | **% Charged amino acids** | **% Hydrophobic amino acids** | **% Polar uncharged amino acids** | **Isoelectric point** |
| --- | --- | --- | --- | --- | --- | --- | --- | --- | --- |
| 1 | ARH11833.1 | 16.38 | 147 | 13.61% | 14.29% | 27.89% | 44.90% | 27.21% | 5.88 |
| 2 | AUR84922.1 | 15.70 | 139 | 12.23% | 17.99% | 30.22% | 45.32% | 27.34% | 8.11 |
| 3 | AUR89477.1 | 16.42 | 145 | 10.34% | 17.93% | 28.28% | 48.97% | 24.83% | 9.84 |
| 4 | AWY10194.1 | 14.23 | 126 | 18.25% | 13.49% | 31.75% | 53.17% | 15.87% | 4.49 |
| 5 | BAV81214.1 | 15.03 | 128 | 14.84% | 17.19% | 32.03% | 43.75% | 27.34% | 6.07 |
| 6 | ASN73021.1 | 9.00 | 82 | 9.76% | 18.29% | 28.05% | 51.22% | 21.95% | 10.49 |
| 7 | AOT26350.1 | 21.73 | 196 | 11.73% | 13.78% | 25.51% | 51.02% | 24.49% | 8.22 |
| 8 | QEG09153.1 _065 | 28.80 | 258 | 15.89% | 10.85% | 26.74% | 46.12% | 27.52% | 4.33 |
| 9 | QJT71317.1 | 19.23 | 171 | 14.62% | 15.79% | 30.41% | 46.20% | 25.15% | 5.82 |
| 10 | ARB13038.1 | 21.73 | 196 | 11.73% | 13.78% | 25.51% | 51.02% | 24.49% | 8.22 |
| 11 | ARB13037.1 | 16.38 | 147 | 13.61% | 14.29% | 27.89% | 44.90% | 27.21% | 5.88 |
| 12 | QEP53420.1 | 21.15 | 182 | 10.44% | 13.74% | 24.18% | 46.70% | 33.52% | 9.03 |
| 13 | AOT26259.1 | 21.73 | 196 | 11.73% | 13.78% | 25.51% | 51.02% | 24.49% | 8.22 |
| 14 | ARH11564.1 | 16.38 | 147 | 13.61% | 14.29% | 27.89% | 44.90% | 27.21% | 5.88 |
| 15 | AGI61769.1 | 19.36 | 174 | 13.22% | 15.52% | 28.74% | 49.43% | 22.41% | 6.19 |
| 16 | QIW90101.1 | 15.93 | 144 | 11.81% | 17.36% | 29.17% | 47.22% | 25.00% | 7.70 |
| 17 | BBI55055.1 | 28.00 | 254 | 14.96% | 10.63% | 25.59% | 49.21% | 25.59% | 4.38 |
| 18 | ARX95207.1 | 21.73 | 196 | 11.73% | 13.78% | 25.51% | 51.02% | 24.49% | 8.22 |
| 19 | ARX95206.1 | 16.38 | 147 | 13.61% | 14.29% | 27.89% | 44.90% | 27.21% | 5.88 |
| 20 | AFV81285.1 | 22.65 | 196 | 9.18% | 14.29% | 23.47% | 48.98% | 32.14% | 9.48 |
| 21 | AUR81381.1 | 17.08 | 150 | 14.00% | 18.00% | 32.00% | 42.67% | 28.00% | 6.34 |
| 22 | AUR81445.1 | 17.19 | 152 | 14.47% | 17.76% | 32.24% | 38.82% | 30.92% | 6.06 |
| 23 | AUR82020.1 | 17.19 | 152 | 14.47% | 17.11% | 31.58% | 38.82% | 31.58% | 5.97 |
| 24 | AUR82085.1 | 18.03 | 158 | 12.66% | 17.09% | 29.75% | 46.20% | 27.22% | 9.21 |
| 25 | AUR82135.1 | 18.03 | 158 | 12.66% | 17.09% | 29.75% | 46.20% | 27.22% | 9.21 |
| 26 | AUR82185.1 | 18.03 | 158 | 12.66% | 17.09% | 29.75% | 46.20% | 27.22% | 9.21 |
| 27 | AUR83594.1 | 17.08 | 150 | 14.00% | 18.00% | 32.00% | 42.67% | 28.00% | 6.34 |
| 28 | AUR83666.1 | 19.51 | 173 | 12.72% | 13.29% | 26.01% | 49.13% | 27.17% | 5.88 |
| 29 | AUR84479.1 | 17.08 | 150 | 14.00% | 18.00% | 32.00% | 42.67% | 28.00% | 6.34 |
| 30 | AUR84996.1 | 17.08 | 150 | 14.00% | 18.00% | 32.00% | 42.67% | 28.00% | 6.34 |
| 31 | AUR85127.1 | 17.08 | 150 | 14.00% | 18.00% | 32.00% | 42.67% | 28.00% | 6.34 |
| 32 | AUR85352.1 | 17.08 | 150 | 14.00% | 18.00% | 32.00% | 42.67% | 28.00% | 6.34 |
| 33 | AUR85483.1 | 17.19 | 152 | 14.47% | 17.11% | 31.58% | 38.82% | 31.58% | 5.97 |
| 34 | AUR86015.11 | 21.91 | 189 | 12.17% | 14.81% | 26.98% | 44.44% | 31.75% | 7.80 |
| 35 | AUR86256.1 | 21.83 | 191 | 10.47% | 17.80% | 28.27% | 48.69% | 25.65% | 9.81 |
| 36 | AUR86475.1 | 15.60 | 139 | 12.23% | 17.99% | 30.22% | 45.32% | 27.34% | 8.11 |
| 37 | AUR86626.1 | 17.28 | 152 | 13.82% | 20.39% | 34.21% | 37.50% | 30.26% | 6.54 |
| 38 | AUR86869.1 | 19.52 | 173 | 12.72% | 13.29% | 26.01% | 47.98% | 28.32% | 5.88 |
| 39 | AUR86942.1 | 19.56 | 173 | 13.29% | 13.29% | 26.59% | 48.55% | 27.17% | 5.45 |
| 40 | AUR87029.1 | 17.19 | 152 | 14.47% | 17.11% | 31.58% | 38.82% | 31.58% | 5.97 |
| 41 | AUR87102.1 | 17.19 | 152 | 14.47% | 17.11% | 31.58% | 38.82% | 31.58% | 5.97 |
| 42 | AUR87803.1 | 18.96 | 177 | 11.30% | 13.56% | 24.86% | 53.67% | 22.03% | 6.34 |
| 43 | AUR88090.1 | 15.70 | 139 | 12.23% | 17.99% | 30.22% | 45.32% | 27.34% | 8.11 |
| 44 | AUR88371.1 | 19.56 | 173 | 13.29% | 13.29% | 26.59% | 48.55% | 27.17% | 5.45 |
| 45 | AUR88640.1 | 15.64 | 139 | 12.23% | 17.99% | 30.22% | 46.04% | 26.62% | 8.26 |
| 46 | AUR88702.1 | 15.57 | 142 | 11.27% | 16.20% | 27.46% | 52.11% | 23.24% | 6.79 |
| 47 | AUR88998.1 | 17.05 | 152 | 13.16% | 14.47% | 27.63% | 48.68% | 25.66% | 6.98 |
| 48 | AUR89640.1 | 17.05 | 152 | 13.16% | 14.47% | 27.63% | 48.68% | 25.66% | 6.98 |
| 49 | AUR89695.1 | 15.68 | 139 | 12.23% | 17.99% | 30.22% | 45.32% | 27.34% | 8.11 |
| 50 | AUR89761.1 | 15.65 | 139 | 12.23% | 17.99% | 30.22% | 46.04% | 26.62% | 8.26 |
| 51 | AUR89891.1 | 15.46 | 139 | 12.23% | 15.83% | 28.06% | 46.76% | 28.06% | 6.67 |
| 52 | AUR90418.1 | 17.05 | 152 | 13.16% | 14.47% | 27.63% | 48.68% | 25.66% | 6.98 |
| 53 | AUR90493.1 | 19.62 | 173 | 13.29% | 13.87% | 27.17% | 47.40% | 27.17% | 5.88 |
| 54 | AUR90549.1 | 15.64 | 139 | 12.95% | 17.27% | 30.22% | 46.76% | 25.90% | 7.57 |
| 55 | AUR91045.1 | 20.64 | 181 | 11.60% | 16.02% | 27.62% | 46.96% | 27.07% | 9.20 |
| 56 | AUR91272.1 | 17.19 | 152 | 15.13% | 17.11% | 32.24% | 38.82% | 30.92% | 5.80 |
| 57 | AUR91696.1 | 15.64 | 139 | 12.95% | 17.27% | 30.22% | 46.76% | 25.90% | 7.57 |
| 58 | AUR91923.1 | 15.71 | 139 | 12.23% | 17.99% | 30.22% | 44.60% | 28.06% | 8.11 |
| 59 | AUR92438.1 | 15.64 | 139 | 12.23% | 17.99% | 30.22% | 46.04% | 26.62% | 8.26 |
| 60 | AUR92674.1 | 17.19 | 152 | 15.13% | 17.11% | 32.24% | 38.82% | 30.92% | 5.80 |
| 61 | AUR93016.1 | 17.26 | 152 | 15.13% | 17.76% | 32.89% | 38.82% | 30.26% | 5.97 |
| 62 | AUR93062.1 | 15.60 | 139 | 12.23% | 17.99% | 30.22% | 46.04% | 26.62% | 8.26 |
| 63 | AUR93136.1 | 20.14 | 184 | 10.87% | 14.67% | 25.54% | 47.28% | 27.17% | 7.39 |
| 64 | AUR93209.1 | 17.95 | 158 | 13.29% | 16.46% | 29.75% | 46.20% | 27.22% | 8.46 |
| 65 | AUR93849.1 | 15.67 | 139 | 12.95% | 17.99% | 30.94% | 45.32% | 26.62% | 7.83 |
| 66 | AUR93915.1 | 15.67 | 139 | 12.95% | 17.99% | 30.94% | 45.32% | 26.62% | 7.83 |
| 67 | AUR93981.1 | 15.67 | 139 | 12.95% | 17.99% | 30.94% | 45.32% | 26.62% | 7.83 |
| 68 | AUR94660.1 | 15.83 | 142 | 12.68% | 12.68% | 25.35% | 50.00% | 26.06% | 5.73 |
| 69 | AUR95425.1 | 15.57 | 142 | 11.27% | 16.20% | 27.46% | 52.11% | 23.24% | 6.79 |
| 70 | AUR96343.1 | 17.16 | 152 | 14.47% | 17.76% | 32.24% | 39.47% | 30.26% | 6.06 |
| 71 | AUR96704.1 | 17.05 | 152 | 13.16% | 14.47% | 27.63% | 48.68% | 25.66% | 6.98 |
| 72 | AUR96787.1 | 17.14 | 152 | 11.84% | 20.39% | 32.24% | 42.76% | 27.63% | 7.86 |
| 73 | AUR96979.1 | 26.19 | 235 | 16.60% | 11.06% | 27.66% | 46.81% | 27.23% | 4.24 |
| 74 | AUR97031.1 | 17.04 | 152 | 14.47% | 13.16% | 27.63% | 48.68% | 25.66% | 4.92 |
| 75 | AUR97524.1 | 15.51 | 139 | 10.79% | 17.27% | 28.06% | 46.76% | 28.06% | 8.38 |
| 76 | AUR97586.1 | 15.68 | 139 | 12.23% | 17.99% | 30.22% | 45.32% | 27.34% | 8.11 |
| 77 | AUR97762.1 | 14.41 | 127 | 13.39% | 14.17% | 27.56% | 51.97% | 21.26% | 6.13 |
| 78 | AUR98485.1 | 19.38 | 177 | 11.86% | 14.12% | 25.99% | 50.85% | 23.73% | 6.19 |
| 79 | AUR98570.1 | 26.19 | 235 | 16.60% | 11.06% | 27.66% | 46.81% | 27.23% | 4.24 |
| 80 | AUR98688.1 | 14.41 | 127 | 13.39% | 14.17% | 27.56% | 51.97% | 21.26% | 6.13 |
| 81 | AUR99421.1 | 17.01 | 152 | 13.82% | 13.82% | 27.63% | 48.03% | 26.32% | 5.57 |
| 82 | AUR99474.1 | 15.60 | 139 | 12.23% | 17.99% | 30.22% | 46.04% | 26.62% | 8.26 |
| 83 | AUS00533.1 | 17.05 | 152 | 12.50% | 13.16% | 25.66% | 49.34% | 26.97% | 6.13 |
| 84 | AUS01456.1 | 19.01 | 177 | 11.86% | 13.56% | 25.42% | 52.54% | 22.60% | 6.08 |
| 85 | AUS01738.1 | 15.78 | 142 | 11.97% | 12.68% | 24.65% | 51.41% | 25.35% | 6.92 |
| 86 | AUS01901.1 | 16.92 | 150 | 13.33% | 17.33% | 30.67% | 42.67% | 29.33% | 6.34 |
| 87 | AUS02126.1 | 17.18 | 152 | 14.47% | 13.16% | 27.63% | 50.66% | 23.68% | 4.93 |
| 88 | AUS02203.1 | 17.18 | 152 | 14.47% | 13.16% | 27.63% | 50.66% | 23.68% | 4.93 |
| 89 | AUS02316.1 | 15.57 | 142 | 11.27% | 16.20% | 27.46% | 52.11% | 23.24% | 6.79 |
| 90 | AJF40822.1 | 21.73 | 196 | 11.73% | 13.78% | 25.51% | 51.02% | 24.49% | 8.22 |
| 91 | AJF40823.1 | 16.38 | 147 | 13.61% | 14.29% | 27.89% | 44.90% | 27.21% | 5.88 |
| 92 | AOT26532.1 | 21.73 | 196 | 11.73% | 13.78% | 25.51% | 51.02% | 24.49% | 8.22 |
| 93 | ARH11595.1 | 16.38 | 147 | 13.61% | 14.29% | 27.89% | 44.90% | 27.21% | 5.88 |
| 94 | AHN84843.1 | 25.33 | 231 | 11.26% | 13.85% | 25.11% | 46.75% | 29.44% | 8.28 |
| 95 | ALP47078.1 | 22.11 | 190 | 9.47% | 18.42% | 27.89% | 45.26% | 30.53% | 10.11 |
| 96 | ALP47458.1 | 22.12 | 190 | 9.47% | 18.95% | 28.42% | 45.26% | 30.00% | 10.17 |
| 97 | QIW88961.1 | 19.19 | 171 | 11.11% | 13.45% | 24.56% | 49.12% | 28.65% | 9.03 |
| 98 | QZI87797.1 | 20.19 | 186 | 10.75% | 14.52% | 25.27% | 47.85% | 26.88% | 7.44 |
| 99 | QZI91501.1 | 20.19 | 186 | 10.75% | 14.52% | 25.27% | 47.85% | 26.88% | 7.44 |
| 100 | QZI91770.1 | 20.19 | 186 | 10.75% | 14.52% | 25.27% | 47.85% | 26.88% | 7.44 |
| 101 | QZI87717.1 | 17.08 | 152 | 12.50% | 19.74% | 32.24% | 42.76% | 27.63% | 7.55 |
| 102 | QZI91091.1 | 17.08 | 152 | 12.50% | 19.74% | 32.24% | 42.76% | 27.63% | 7.55 |
| 103 | QZI91807.1 | 20.19 | 186 | 10.75% | 14.52% | 25.27% | 47.85% | 26.88% | 7.44 |
| 104 | QZI92121.1 | 20.19 | 186 | 10.75% | 14.52% | 25.27% | 47.85% | 26.88% | 7.44 |
| 105 | QZI91464.1 | 20.19 | 186 | 10.75% | 14.52% | 25.27% | 47.85% | 26.88% | 7.44 |
| 106 | QZI92038.1 | 20.19 | 186 | 10.75% | 14.52% | 25.27% | 47.85% | 26.88% | 7.44 |
| 107 | QZI91999.1 | 20.19 | 186 | 10.75% | 14.52% | 25.27% | 47.85% | 26.88% | 7.44 |
| 108 | QZI91303.1 | 20.19 | 186 | 10.75% | 14.52% | 25.27% | 47.85% | 26.88% | 7.44 |
| 109 | QZI91264.1 | 20.19 | 186 | 10.75% | 14.52% | 25.27% | 47.85% | 26.88% | 7.44 |
| 110 | QZI91180.1 | 17.07 | 152 | 11.84% | 19.74% | 31.58% | 43.42% | 27.63% | 7.85 |
| 111 | QZI92139.1 | 18.02 | 158 | 12.66% | 16.46% | 29.11% | 46.20% | 27.85% | 8.86 |
| 112 | QZI91929.1 | 15.35 | 134 | 11.94% | 21.64% | 33.58% | 46.27% | 22.39% | 10.15 |
| 113 | QZI91403.1 | 15.35 | 134 | 11.94% | 21.64% | 33.58% | 46.27% | 22.39% | 10.15 |
| 114 | QZI91609.1 | 17.07 | 152 | 11.84% | 19.74% | 31.58% | 43.42% | 27.63% | 7.85 |
| 115 | QZI91719.1 | 17.07 | 152 | 11.84% | 19.74% | 31.58% | 43.42% | 27.63% | 7.85 |
| 116 | AOT26714.1 | 21.73 | 196 | 11.73% | 13.78% | 25.51% | 51.02% | 24.49% | 8.22 |
| 117 | ARH11656.1 | 16.38 | 147 | 13.61% | 14.29% | 27.89% | 44.90% | 27.21% | 5.88 |
| 118 | AHJ87874.1 | 19.42 | 171 | 13.45% | 14.04% | 27.49% | 47.95% | 26.90% | 5.89 |
| 119 | AOT26623.1 | 21.73 | 196 | 11.73% | 13.78% | 25.51% | 51.02% | 24.49% | 8.22 |
| 120 | ARH11626.1 | 16.38 | 147 | 13.61% | 14.29% | 27.89% | 44.90% | 27.21% | 5.88 |
| 121 | AQT28060.1 | 24.48 | 210 | 12.38% | 15.71% | 28.10% | 48.10% | 27.62% | 8.24 |
| 122 | ARB13293.1 | 21.73 | 196 | 11.73% | 13.78% | 25.51% | 51.02% | 24.49% | 8.22 |
| 123 | ARB13292.1 | 16.38 | 147 | 13.61% | 14.29% | 27.89% | 44.90% | 27.21% | 5.88 |
| 124 | ARB13384.1 | 21.73 | 196 | 11.73% | 13.78% | 25.51% | 51.02% | 24.49% | 8.22 |
| 125 | ARH11821.1 | 15.95 | 143 | 13.99% | 14.69% | 28.67% | 43.36% | 27.97% | 5.88 |
| 126 | APC46076.1 | 21.73 | 196 | 11.73% | 13.78% | 25.51% | 51.02% | 24.49% | 8.22 |
| 127 | APC46056.1 | 16.38 | 147 | 13.61% | 14.29% | 27.89% | 44.90% | 27.21% | 5.88 |
| 128 | AOT26896.1 | 21.73 | 196 | 11.73% | 13.78% | 25.51% | 51.02% | 24.49% | 8.22 |
| 129 | ARH11718.1 | 16.38 | 147 | 13.61% | 14.29% | 27.89% | 44.90% | 27.21% | 5.88 |
| 130 | AOT27174.1 | 21.73 | 196 | 11.73% | 13.78% | 25.51% | 51.02% | 24.49% | 8.22 |
| 131 | ARH11893.1 | 16.38 | 147 | 13.61% | 14.29% | 27.89% | 44.90% | 27.21% | 5.88 |
| 132 | ARB13475.1 | 21.73 | 196 | 11.73% | 13.78% | 25.51% | 51.02% | 24.49% | 8.22 |
| 133 | ARB13474.1 | 16.38 | 147 | 13.61% | 14.29% | 27.89% | 44.90% | 27.21% | 5.88 |
| 134 | QQM14055.1 | 18.30 | 164 | 13.41% | 15.24% | 28.66% | 46.95% | 26.22% | 6.07 |
| 135 | ARM71055.1 | 19.35 | 173 | 14.45% | 15.03% | 29.48% | 46.82% | 23.70% | 5.69 |
| 136 | AII27511.1 | 28.23 | 254 | 16.54% | 11.02% | 27.56% | 48.03% | 24.80% | 4.26 |
| 137 | APU00145.1 | 14.80 | 129 | 10.85% | 13.95% | 24.81% | 43.41% | 34.11% | 6.34 |
| 138 | ALM62052.1 | 25.90 | 236 | 13.56% | 13.98% | 27.54% | 47.88% | 26.69% | 5.92 |
| 139 | QGH73788.1 | 21.91 | 189 | 8.99% | 17.46% | 26.46% | 49.21% | 25.40% | 10.14 |
| 140 | AIA10315.1 | 25.36 | 231 | 11.26% | 13.85% | 25.11% | 46.32% | 29.87% | 8.28 |
| 141 | AIA10322.1 | 11.58 | 103 | 14.56% | 12.62% | 27.18% | 50.49% | 22.33% | 4.80 |
| 142 | AJF40686.1 | 19.38 | 174 | 13.79% | 14.94% | 28.74% | 49.43% | 22.41% | 5.76 |
| 143 | QZI93087.1 | 15.61 | 138 | 15.94% | 19.57% | 35.51% | 46.38% | 19.57% | 5.98 |
| 144 | QZI93040.1 | 15.61 | 138 | 15.94% | 19.57% | 35.51% | 46.38% | 19.57% | 5.98 |
| 145 | QZI92941.1 | 15.61 | 138 | 15.94% | 19.57% | 35.51% | 46.38% | 19.57% | 5.98 |
| 146 | QZI92815.1 | 15.61 | 138 | 15.94% | 19.57% | 35.51% | 46.38% | 19.57% | 5.98 |
| 147 | QZI92574.1 | 15.56 | 138 | 15.22% | 19.57% | 34.78% | 47.10% | 19.57% | 6.13 |
| 148 | QZI86512.1 | 15.61 | 138 | 15.94% | 19.57% | 35.51% | 46.38% | 19.57% | 5.98 |
| 149 | QZI87835.1 | 16.23 | 141 | 14.89% | 20.57% | 35.46% | 43.97% | 24.82% | 7.19 |
| 150 | QZI87890.1 | 16.23 | 141 | 14.89% | 20.57% | 35.46% | 43.97% | 24.82% | 7.19 |
| 151 | QZI89516.1 | 19.03 | 177 | 11.86% | 13.56% | 25.42% | 51.98% | 23.16% | 5.96 |
| 152 | AFE86379.1 | 14.53 | 125 | 9.60% | 16.80% | 26.40% | 48.00% | 29.60% | 10.25 |
| 153 | AOT27083.1 | 21.73 | 196 | 11.73% | 13.78% | 25.51% | 51.02% | 24.49% | 8.22 |
| 154 | ARH11778.1 | 16.38 | 147 | 13.61% | 14.29% | 27.89% | 44.90% | 27.21% | 5.88 |
| 155 | AOT26990.1 | 21.60 | 196 | 11.22% | 14.29% | 25.51% | 52.04% | 23.47% | 8.82 |
| 156 | ARH11749.1 | 16.43 | 147 | 12.24% | 13.61% | 25.85% | 43.54% | 30.61% | 6.98 |
| 157 | AVR75885.1 | 17.23 | 152 | 14.47% | 17.11% | 31.58% | 49.34% | 21.05% | 7.03 |
| 158 | AFF27942.1 | 28.74 | 258 | 8.91% | 13.18% | 22.09% | 41.86% | 37.60% | 8.08 |
| 159 | QNJ54818.1 | 22.11 | 190 | 9.47% | 18.42% | 27.89% | 45.26% | 30.53% | 10.11 |
| 160 | QNJ55205.1 | 22.12 | 190 | 9.47% | 18.95% | 28.42% | 45.26% | 30.00% | 10.17 |
| 161 | AFC22744.1 | 17.47 | 154 | 13.64% | 14.94% | 28.57% | 48.70% | 24.68% | 6.36 |
| 162 | AGI61845.1 | 19.36 | 174 | 13.22% | 15.52% | 28.74% | 49.43% | 22.41% | 6.19 |
| 163 | ALY07056.1 | 19.86 | 179 | 10.61% | 15.08% | 25.70% | 49.72% | 24.58% | 9.24 |
| 164 | ACR16491.1 | 15.91 | 144 | 11.81% | 17.36% | 29.17% | 47.92% | 24.31% | 7.70 |
| 165 | AFH14429.1 | 15.98 | 144 | 11.81% | 18.06% | 29.86% | 47.22% | 24.31% | 7.71 |
| 166 | AGN34270.1 | 15.67 | 144 | 7.64% | 14.58% | 22.22% | 50.00% | 29.17% | 10.15 |
| 167 | AGW43571.1 | 21.37 | 195 | 9.74% | 11.79% | 21.54% | 49.23% | 30.77% | 7.89 |
| 168 | AUM58751.1 | 22.79 | 196 | 9.18% | 13.27% | 22.45% | 49.49% | 33.16% | 9.44 |


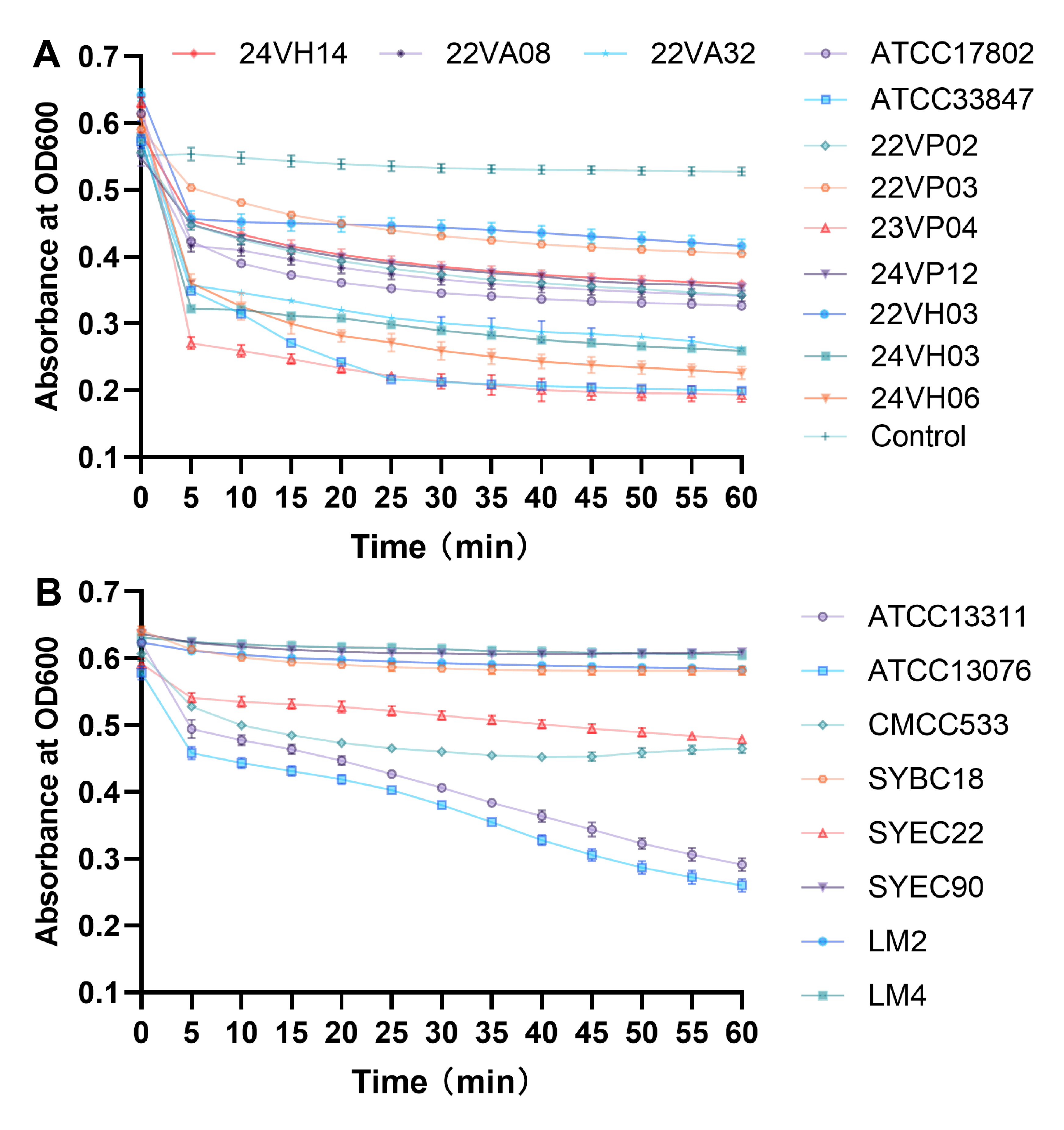


**FIG S1 Synergistic bactericidal kinetics of LysMD30 combined with colistin.** (A) 12 Vibrio strains (six V. parahaemolyticus, four V. harveyi, and two V. alginolyticus strains); (B) Three Salmonella strains, three *E. coli* strains, and two L. monocytogenes strains.

**References**

[1] CHAIKEERATISAK V. Development of a novel phage-derived endolysin as an outer membrane-penetrating antibacterial against bacterial pathogens in aquaculture[J]. Report of Grant-Supported Research The Asahi Glass Foundation, 2024, 92.

[2] Xia H, Yang H, Yan N *et al*. Bacteriostatic effects of phage F23s1 and its endolysin on *Vibrio parahaemolyticus*[J]. J Basic Microbiol, 2022, 62(8):963-974.

[3] Ning HQ, Lin H, Wang JX. Synergistic effects of endolysin Lysqdvp001 and epsilon-poly-lysine in controlling *Vibrio parahaemolyticus* and its biofilms[J]. Int J Food Microbiol, 2021, 343:109112.

[4] Chen J, Zhao Z, Mu X *et al*. Characterization of a marine endolysin LysVPB against *Vibrio parahaemolyticus*[J]. Protein Expr Purif, 2025, 226:106608.

[5] Melo-Lopez FN, Zermeno-Cervantes LA, Barraza A et al. Biochemical characterization of LysVpKK5 endolysin from a marine *vibrio* phage[J]. Protein Expr Purif, 2021, 188:105971.

[6] Matamp N, Bhat SG. Phage endolysins as potential antimicrobials against multidrug resistant *Vibrio alginolyticus* and *Vibrio parahaemolyticus*: current status of research and challenges ahead[J]. Microorganisms, 2019, 7(3).

[7] Li M, Jin Y, Lin H *et al*. Complete Genome of a novel lytic *Vibrio parahaemolyticus* phage VPp1 and characterization of its endolysin for antibacterial activities[J]. J Food Prot, 2018, 81(7):1117-1125.

[8] Liu J, Wu Q, Malakar PK *et al*. Mining and multifaceted applications of phage lysin for combatting *Vibrio parahaemolyticus*[J]. Food Res Int, 2024, 192:114819.

[9] Lim J-A, Lee N, Chun H-S *et al*. Characterization of a novel endolysin from bacteriophage infecting *Vibrio parahaemolyticu*s, vB_VpaP_KF2[J]. Applied Biological Chemistry, 2020, 63(1).

[10] Li X, Zhang C, Wei F *et al*. Bactericidal activity of a holin-endolysin system derived from *Vibrio alginolyticus* phage HH109[J]. Microb Pathog, 2021, 159:105135.
